# The glycointeractome of serogroup B *Neisseria meningitidis* strain MC58

**DOI:** 10.1038/s41598-017-05894-w

**Published:** 2017-07-18

**Authors:** Tsitsi D. Mubaiwa, Lauren E. Hartley-Tassell, Evgeny A. Semchenko, Freda. E.-C. Jen, Yogitha N. Srikhanta, Christopher J. Day, Michael P. Jennings, Kate L. Seib

**Affiliations:** 1 Institute for Glycomics, Griffith University, Gold Coast, Queensland Australia; 2 Biomedicine Discovery Institute, Department of Microbiology, Monash University, Clayton, Victoria Australia

## Abstract

*Neisseria meningitidis* express numerous virulence factors that enable it to interact with diverse microenvironments within the host, during both asymptomatic nasopharyngeal colonization and invasive disease. Many of these interactions involve bacterial or host glycans. In order to characterise the meningococcal glycointeractome, glycan arrays representative of structures found on human cells, were used as a screening tool to investigate host glycans bound by *N. meningitidis*. Arrays probed with fluorescently labelled wild-type MC58 revealed binding to 223 glycans, including blood group antigens, mucins, gangliosides and glycosaminoglycans. Mutant strains lacking surface components, including capsule, lipooligosaccharide (LOS), Opc and pili, were investigated to identify the factors responsible for glycan binding. Surface plasmon resonance and isothermal calorimetry were used to confirm binding and determine affinities between surface components and host glycans. We observed that the L3 LOS immunotype (whole cells and purified LOS) bound 26 structures, while L8 only bound 5 structures. We further demonstrated a direct glycan-glycan interaction between purified L3 LOS and Thomsen–Friedenreich (TF) antigen, with a K_D_ of 13 nM. This is the highest affinity glycan-glycan interaction reported to date. These findings highlight the diverse glycointeractions that may occur during different stages of meningococcal disease, which could be exploited for development of novel preventative and therapeutic strategies.

## Introduction

*Neisseria meningitidis* is an exclusively human pathogen that poses a considerable public health threat. Of the 13 meningococcal capsular serogroups described, six (A, B, C, W, X, Y) are associated with the majority of disease^1^. Capsular polysaccharide based vaccines against serogroups A, C, W and Y, and an outer membrane protein based vaccine for serogroup B are available^2^. These vaccines are not universally administered and there is no serogroup X vaccine, leaving several populations susceptible to meningococcal disease. Despite the availability of effective antibiotics, the difficulty in diagnosis and the rapid progression of meningococcal disease may result in delayed treatment. As a result, *N. meningitidis* remains a leading cause of bacterial meningitis and sepsis^3^, with mortality rates as high as 10% and survivors often endure debilitating sequelae^4^. Furthermore, as the worldwide circulation of serogroups continues to change^4^, there is an increasing need for a broad spectrum vaccine. As such, a better understanding of meningococcal host pathogen interactions is vital.

*N. meningitidis* expresses numerous surface components that allow it to interact with and survive within diverse microenvironments in the host. These interactions result in different pathologies, ranging from asymptomatic nasopharyngeal colonization of the airway epithelia, to invasive disease that manifest as sepsis and meningitis^4^. Carbohydrate structures (glycans) on the bacterium, and on host cells, play a key role in these processes. For example, the polysaccharide capsule of *N. meningitidis* is crucial for invasive disease, providing protection from innate and adaptive immune responses, and isolates from the blood and cerebrospinal fluid are invariably encapsulated^1^. In conjunction with the capsule, lipooligosaccharide (LOS) modulates host-pathogen interactions^5^ and is implicated in virulence. Meningococcal LOS is made up of two oligosaccharide chains attached to heptose residues^6^, and the differences in the composition of these oligosaccharides form the basis of meningococcal immunotyping (L1-L12)^7^. The LOS of *Neisseria gonorrhoeae* has been shown to mediate direct adherence to the host, binding to the asialoglycoprotein receptor on epithelial cells^8^, but a similar role for meningococcal LOS has not been investigated.

Several major meningococcal protein adhesins such as pili (type IV fimbriae) and the opacity protein, Opc, have interactions that are dependent on host glycans. Meningococcal pili are macromolecular heteropolymeric proteins that can be glycosylated, and which protrude past the polysaccharide capsule and promote meningococcal adherence to endothelial and epithelial host cells^9^. The binding of this key adhesin to the platelet activating factor receptor on host cells is influenced by the pilin-linked glycan^10^. The opacity protein, Opc, is a phase variable, integral, β-barrel outer membrane protein, the expression of which results in agar-grown colonies having an opaque phenotype. Opc is involved in meningococcal adherence^9, 11^ and invasion^5, 11, 12^. Opc interacts with human extracellular matrix glycans such the heparan sulfate proteoglycans^13^ and the glycoproteins fibronectin and vitronectin, mediating meningococcal adherence and invasion into endothelial cells^11^. However, not all host cell target receptors for pili and Opc may be known.

The human niches that *N. meningitidis* encounters within the host are all highly glycosylated. This, coupled with the fact that many bacterial infections of the human host rely on glycan based interactions^14^, indicates that further investigation of *N. meningitidis* glycan interactions may uncover new aspects of its pathobiology and inform new strategies for the prevention and treatment of disease. To date, the glycointeractome of *N. meningitidis* has not been studied in a systematic manner. Here we have employed glycan array analysis to identify and characterise the glycointeractome of a serogroup B strain of *N. meningitidis*, strain MC58.

## Results

### Glycan array analysis reveals that *Neisseria meningitidis* binds to numerous glycans from different structural and functional classes

**Figure 1:**
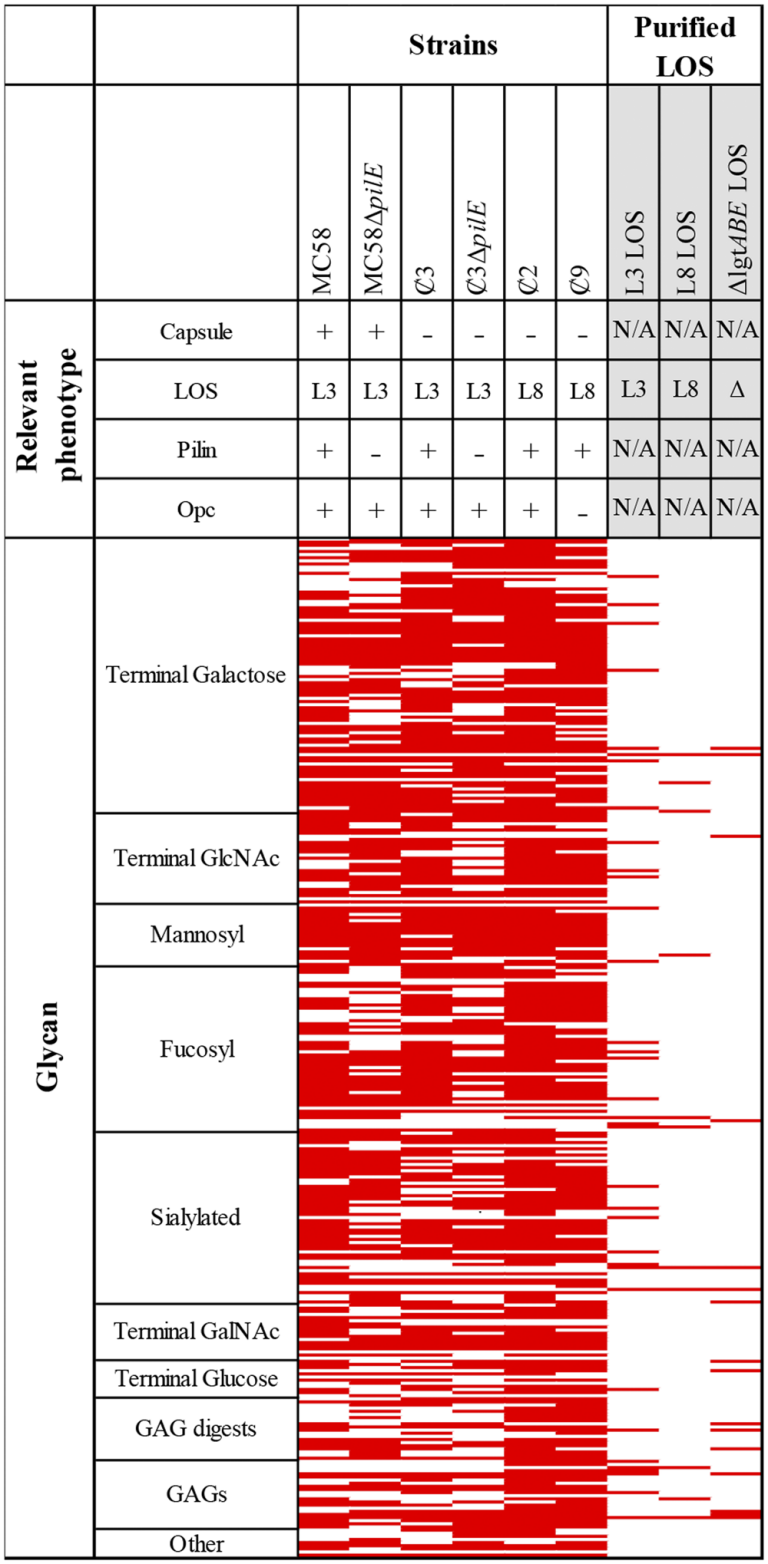
Heat map showing glycan binding by *N. meningitidis* from glycan array analysis of *N. meningitidis* wild-type and mutants lacking key outer membrane components. Red represents binding to the glycan structure (in three independent replicates) and white is no binding observed. Glycans are grouped based on terminating structures/ monosaccharide composition. For the full list of binding to individual glycans see Supplementary Table S1. GAG, Glycosaminoglycan; GalNAc, *N*-acetylgalactosamine; GlcNAc, *N*-acetylglucosamine.

In order to characterise glycan binding by serogroup B *N*. *meningitidis*, glycan array technology was used as a screening tool^15^. The encapsulated serogroup B *N. meningitidis* strain MC58 was fluorescently labelled and incubated on glycan arrays to detect binding. The glycan arrays were printed with 367 glycan structures representative of those found on human cells. The glycans include those with different chemical structures and biological functions. Wild-type MC58 bound to 223 glycans on the array, including fucosylated, sialylated, mannosylated and glycosaminoglycan (GAG) structures, as well as glycans displaying terminal galactose (Gal), *N*-acetylgalactosamine (GalNAc) or terminal *N*-acetylglucosamine (GlcNAc) groups (Fig. 1 and Supplementary Table S1). Bound glycan structures are associated with a range of different biological functions, and included blood group antigens, gangliosides and mucin O-glycans.

### Glycan array analysis of mutant strains to identify the meningococcal surface components mediating glycan binding

**Figure 2:**
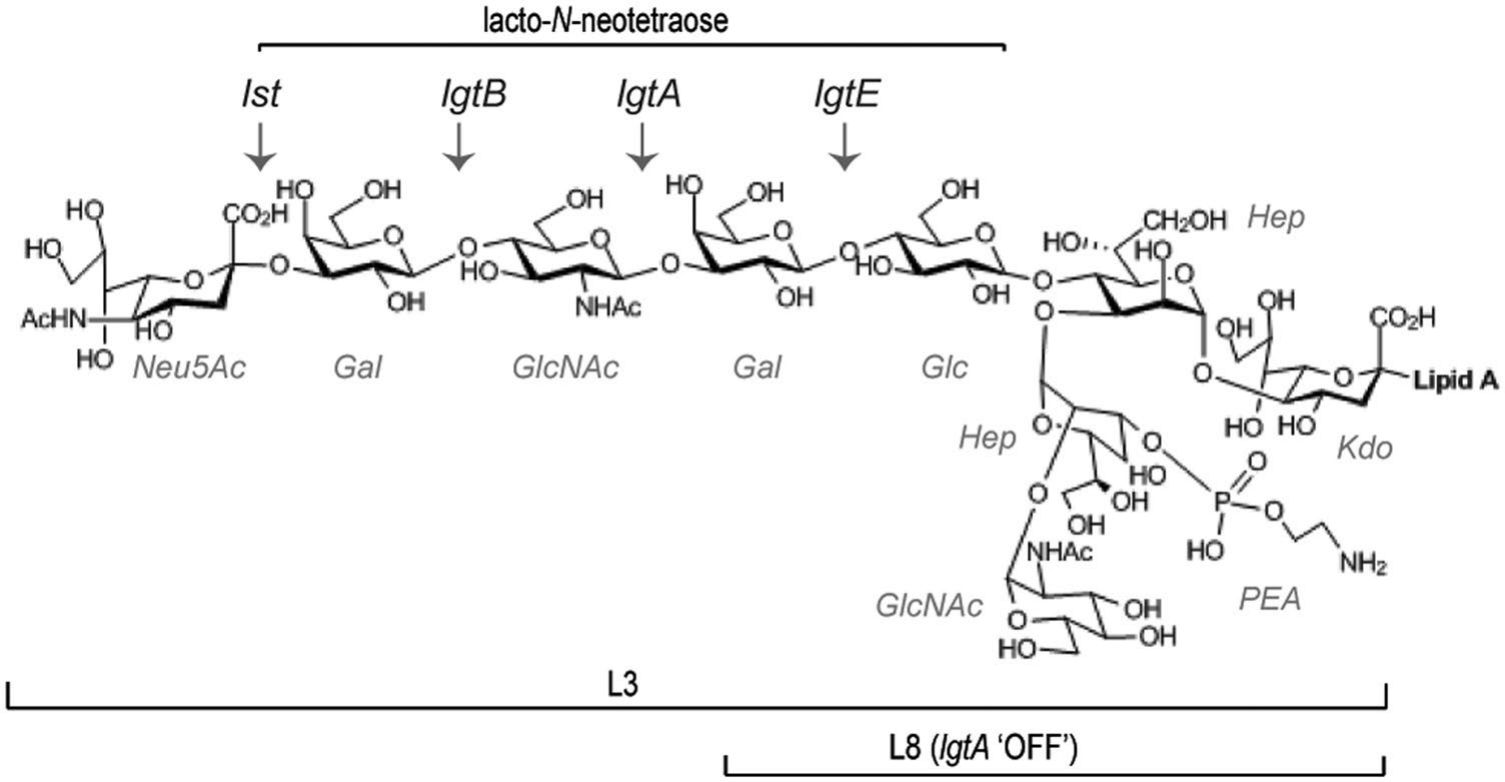
Chemical structure of LOS from *N. meningitidis* MC58. Phase variable glycosyltransferase genes involved in LOS biosynthesis are shown (*lst, lgtB*, *lgtA, lgtE*), and arrows indicate the sugars added by these enzymes. The structures of the L3 and L8 immunotypes are indicated. L8 immunotype is expressed when *lgtA* is phase varied off. The terminal lacto-*N*-neotetraose epitope of L3 LOS is shown.

To investigate the meningococcal surface components potentially responsible for the glycan binding observed by the MC58 wild-type strain, a panel of isogenic mutants or phase variants each lacking a key outer membrane component were also analysed in three independent replicates (see Table 1 for strains used). Mutant strains included those lacking; polysaccharide capsule, pili and Opc, as well as natural variants that express either the L3 or L8 LOS immunotypes (Fig. 2). Figure 1 shows that when a key surface component was deleted, several interactions previously seen with the wild-type MC58 were lost (Supplementary Table S1). In some cases, binding to new structures was also observed, suggesting that the relevant outer membrane component was potentially blocking interactions. This was evident for both the non-encapsulated mutant and the LOS variants, where binding to specific glycans was both lost and gained relative to the MC58 wild-type profile.

**Table 1:** Strains used in this study.

Strain	Relevant phenotype	Source/Ref
MC58 wild-type	Cap + , L3^v^, pilin^v^ + , Opc +	52
ȼ2	Cap−, L8^v^, pilin^v^ + , Opc+	5
ȼ3	Cap−, L3^v^, pilin^v^+, Opc+	5
ȼ4	Cap−, L3^v^, pilin^v^−, Opc +	5
ȼ9	Cap−, L8^v^, pilin^v^+, Opc−	5
ȼ11	Cap−, L3^v^, pilin^v^−, Opc−	5
ȼ3∆*lst*	Cap−, L3^v^, pilin^v^ + , Opc +	This study
ȼ3∆*lgtABE*	Cap−, L8^v^, pilin^v^ + , Opc+	17
MC58∆*pilE*	Cap+, L3^v^, pilin−, Opc+	This study
ȼ3∆*pilE*	Cap−, L3^v^, pilin−, Opc+	This study

^**v**^ = natural phase variant. All strains used in this study are natural LOS variants. Non-encapsulated mutants were created by insertion of an erythromycin cassette into the polysialyltransferase gene (*siaD*)^5^. Opc mutants were created by insertion of an kanamycin cassette into the Opc gene^5^. Strains MC58∆*pilE* and Ȼ3∆*pilE* are *pilE* knockout mutants created by transformation of MC58 and Ȼ3, respectively, with a *pilE* construct from *N. meningitidis* C311*#3* that has a kanamycin resistance cassette in *pilE* ^45^.

#### Capsule modulates meningococcal glycointeractions

When compared to MC58 wild-type, the capsule knockout strain Ȼ3 lost binding to 40 glycans and bound 43 additional structures. While both MC58 wild-type and Ȼ3 bound glycans from all classes tested (Fig. 1), they differed in their ability to recognise blood group antigens. Unlike the MC58 wild-type, Ȼ3 bound to group A antigen structures (blood group A disaccharide (Index Number (_IN_) _IN_102 in Supplementary Table S1) and A type 2 (_IN_368)). Conversely, the MC58 wild-type did not bind blood group A structures, it uniquely bound the linear B-2 trisaccharide (_IN_1O) and H type 2 (_IN_216) antigen.

#### Glycointeractions mediated by pili

The role of pili in host-pathogen interactions has been described for both the encapsulated and non-encapsulated meningococci. Taking this into consideration, two pili mutants, one with and one without capsule, were used to investigate pili-based glycointeractions. When comparing glycan interactions of the encapsulated, wild-type MC58 and pili mutant (MC58Δ*pilE*), 56 interactions were lost. Similarly, comparison of the non-encapsulated pili mutant (Ȼ3Δ*pilE*) with the non-encapsulated isogenic parent strain showed that a total of 61 interactions were lost.

The absence of pili in either the encapsulated or non-encapsulated background resulted in loss of binding to a similar sub-set of structures on the glycan array, however there were differences related to charge of the glycan. For example, the non-encapsulated strain Ȼ3Δ*pilE* lost binding to the asialo GM1 (_IN_382), while the encapsulated strain MC58Δ*pilE* lost binding to the GD2 ganglioside (_IN_531). Both glycan structures contain a core GalNAcβ1-4Galβ1-4Glc structure, but asialo GM1 has a terminal galactose, while GD2 has a terminal sialic acid that gives it a negative charge. A similar charge effect was observed with several other glycans, indicating that the anionic polysialic acid capsule is interfering with these interactions.

#### Glycointeractions mediated by Opc

A comparison of the glycan binding profile of strain Ȼ2 (Opc+) with strain Ȼ9 (Opc−) revealed a loss of binding to 43 glycans. The interactions that were lost included several terminal Gal structures that contained a lactose (Galβ1-4Glc) and/or N-acetyllactosamine (Galβ1-4GlcNAc) epitope. Binding to many blood group antigens and sialylated glycans was also lost, including; H disaccharide (_IN_1H), sialyl LewisX (_IN_10B), 3′-sialyllactose (_IN_11A) & 6′-sialyllactose (_IN_11B). The Opc mutant (Ȼ9) also lost binding to the GAG chondroitin sulfate (_IN_13M) and a disaccharide of chondroitin sulfate; delta di-disD (_IN_13B).

#### Glycointeractions mediated by LOS

*N. meningitidis* strain Ȼ2 expresses L8 immunotype LOS (Fig. 2), and bound to 269 structures on the glycan array, while the L3 immunotype LOS expressing strain (Ȼ3) bound 225 structures. Both strains bound to a common set of 208 glycans, while 61 glycan structures were only bound by the L8 strain, and 17 were only bound by the L3 strain. The glycans bound only by the L8 strain are mostly GAGs and sialylated glycans, including chondroitin sulfate, heparinase digests, sialylated blood group antigens, sialyl LewisA (_IN_10A) and sialyl LewisX (_IN_10B) and the 3′-sialyllactose (_IN_11A) and 6′-sialyllactose (_IN_11B) lactosyl glycans.

### Detailed analysis of Opc and LOS mediated glycointeractions

To fully characterise the glycan interactions revealed above, we used purified meningococcal surface components to investigate their direct binding to host glycans, and to quantitate binding affinities. Here we describe detailed analysis of two meningococcal surface components; Opc and LOS.

#### Affinity determination of Opc-mediated glycan binding

Opc-mediated glycan binding was further investigated using outer membrane preparations from the Opc expressing strain (Ȼ4) and the isogenic Opc mutant strain (Ȼ11). Isothermal titration calorimetry (ITC) was used to confirm interactions and quantify binding affinities of five glycans, chosen from the 43 glycan interactions putatively ascribed to Opc based on the glycan array analysis of the wild-type and Opc mutant strains (above). Five of the interactions tested were high affinity, with a dissociation constant (K_D_) below 5 µM (Table 2, Supplementary data S1). Consistent with previous reports^16^, Opc bound 2,6 sialylactose with a K_D_ of ~1 µM (713 nM) and sialyl LewisX (K_D_ ~590 nM). Binding was also observed to chondroitin-6-sulfate polymer (K_D_ ~630 nM) and lacto-*N*-neotetraose (K_D_ = 1.765 µM). Opc binding to these ligands has not been previously reported.

**Table 2:** ITC analysis of Opc-glycan interactions.

Glycan			K_D_ (μM)
Index	Name	Structure	
10B	sialyl LewisX	Neu5Acα2-3Galβ1-4(Fucα1-3)GlcNAc	0.593 ± 0.17
11B	2,6′-sialyllactose	Neu5Acα2-6Galβ1-4Glc	0.713 ± 0.14
71	blood group H disaccharide	Fucα1-2Galβ	2.950 ± 0.59
383	lacto-*N*-neotetraose	Galβ1-4GlcNAcβ1-3Galβ1-4Glcβ	1.765 ± 0.69
13 M	Chondroitin-6-sulfate	(GlcA/IdoAβ1-3( ± 6 S)GalNAcβ1-4)n (n < 250)	0.628 ± 0.16

Opc-glycan interactions were determined by comparison of outer membrane preparations from the Opc+Ȼ4 and the Opc− Ȼ11 strains (Table 1). Refer to Supplementary data S1 for sensorgrams for each individual experiment.

#### Affinity determination of LOS-mediated glycan binding

To examine direct LOS-mediated binding to glycans in the absence of other surface components, glycan array studies were conducted using purified, fluorescently labelled LOS from Ȼ3 (L3 immunotype LOS), Ȼ2 (L8 immunotype LOS) and Ȼ3Δ*lgtABE* (L8 LOS structure that lacks the terminal galactose residue) (Fig. 2). Purified L3 LOS bound to 35 glycan structures on the array, L8 LOS bound to 11 and the truncated L8 LOS bound to 16. These interactions are ascribed to direct binding of the LOS to glycan structures on the array. Glycans bound by L3 and L8 LOS types were mutually exclusive, except for hyaluronan (_IN_14H), which was bound by both L3 and L8 LOS. L3 LOS bound short-chain glycans displaying a terminal galactose, *e.g*. lacto-*N*-biose I (_IN_1A), the Thomsen–Friedenreich (TF) antigen (_IN_1E), and large chain fucosylated glycans containing an *N*-acetyllactosamine (Galβ1-4GlcNAc) epitope, including lacto-*N*-fucopentaose III (_IN_7C). L3 LOS also bound a α2-8 linked sialic acid trisaccharide (_IN_321) and the GAG, heparin (_IN_13J). Unlike L3 LOS, purified L8 LOS did not bind any glycans with terminal GlcNAc or glucose.

Five glycans were selected for further investigation by surface plasmon resonance (SPR), to confirm LOS-glycan interactions using a different analytical technique and to quantify the binding affinities (Table 3). These glycans were selected to represent the different classes of glycans bound, and included those of potential importance in meningococcal pathogenesis. All five glycans were bound by both the L8 and L3 expressing strains on the glycan array, and included three that the purified L3 LOS also bound to on the glycan array (lacto-*N*-biose I, TF antigen, heparin), one that purified L8 LOS bound (α1-3 galactobiose), and one the truncated L8 LOS from Ȼ3Δ*lgtABE* bound (colominic acid). SPR revealed that L8 bound three of the glycans tested, while L3 LOS bound all five of the glycans. The highest affinity interaction detected was between L3 LOS and the TF antigen (K_D_ = 13 nM ± 2 nM) (Table 3). This is the highest affinity glycan-glycan interaction reported to date.

**Table 3:** SPR analysis of LOS mediated glycointeractions.

Glycan		K_D_ (µM)			
Index	Name	L3	Δ*lst*	L8	Δ*lgtABE*
1 A	lacto-*N*-biose I	1.27 ± 0.54	2.76 ± 0.44	N	N
1E	TF antigen	0.013 ± 0.002	0.033 ± 0.016	N	N
1 N	α1-3 galactobiose	1.37 ± 0.11	0.51 ± 0.07	0.543 ± 0.09	N
11 C	colominic acid	0.320 ± 0.139	0.29 ± 0.137	0.42 ± 0.066	0.369 ± 0.185
13 J	heparin	0.454 ± 0.202	0.53 ± 0.11	0.456 ± 0.037	0.489 ± 0.14

N: No concentration dependent binding observed within the instrument’s detection range.

L3, Δ*lst*, L8 and Δ*lgtABE* refer to lipooligosaccharide (LOS) structures purified from MC58 wild-type, Ȼ3, Ȼ3Δ*lst*, and Ȼ3Δ*lgtABE* strains, respectively (Table 1 and Fig. 2). Refer to Supplementary data S2 for sensorgrams for each individual experiment.

L8 LOS is a truncated version of the L3 LOS. L8 LOS lacks the terminal lacto-*N*-neotetraose structure of L3 LOS due to the phase variation of the LgtA glycosyltransferase that generates switching between these LOS immunotypes^17^ (Fig. 2). Comparison of the binding of these LOS immunotypes may indicate the exact regions of LOS that is responsible for the observed interactions with host glycans. To further define the minimum LOS structure required for binding, we used LOS purified from two well-characterised LOS mutant strains^18^. Ȼ3Δ*lst* expresses an L3 LOS structure that lacks the terminal Neu5Ac residue, and Ȼ3Δ*lgtABE* expresses an L8 LOS structure that lacks the terminal galactose residue (Fig. 2). The data in Table 3 indicates that the terminal Neu5Ac residue of L3 LOS is not essential for the binding to these structures. The terminal galactose of the L8 LOS was required for binding to α1-3 galactobiose (_IN_1N), but not for binding to heparin (_IN_13J) and colominic acid (_IN_11C). All four LOS structures bound heparin and colominic acid via high affinity interactions indicating that the Ȼ3Δ*lgtABE* LOS structure defines the minimum structure required for these interactions.

## Discussion

Meningococcal niches within the human host are highly glycosylated environments. These microenvironments contain GAGs that are commonly exploited by bacterial pathogens for adherence and invasion^19–21^. The nasopharyngeal epithelia has abundant mucus and mucins, which are made up of up to 90% glycan^22^. The central nervous system is also an extremely glycan rich environment, with gangliosides^23^ and GAGs such as chondroitin sulfate present^24^. Association with host glycans has been reported in several studies of meningococcal virulence factors (including Opc^13, 16^, Opa^13, 16^, and NHBA^25, 26^), but systematic, omics approaches have not be used and there is potential for further interactions between *N. meningitidis* and host glycans to be discovered. In the current study, we sought to characterise the glycointeractome of *N. meningitidis*.

Using the high throughput method of glycan array analysis, we have shown that *N. meningitidis* binds multiple host glycans. Glycan array technology is a novel qualitative method that is relatively new to molecular microbiology. Glycan arrays contain a large number of glycans that are immobilised onto a solid support, and fluorescently labelled ligands are incubated onto the slides to allow binding and subsequent visualization^27, 28^. This technique has been used to identify and investigate interactions between several different microbes and host glycans^15, 29–31^.

Previous studies have reported glycan binding by several *N. meningitidis* surface expressed virulence factors^13, 16, 25^. Of the interactions revealed in this study (Fig. 1), approximately 30% can be explained by what is currently known about meningococcal glycan interactions. For example, binding to sialylated monosaccharides and heparin residues may be attributed to the Opacity proteins^16^. Heparin binding may also be attributed to the Neisseria heparin binding antigen (NHBA)^25, 26^. Our new data reveals novel interactions between the meningococcus and host glycans. Binding to mannosyl structures, complex galactose structures and hyaluronan glycosaminoglycans by *N. meningitidis* has not been reported previously, and the surface components responsible for mediating these interactions are unknown. Glycan array analysis of a series of isogenic mutants allowed us to putatively ascribe these new glycan interactions to specific meningococcal surface components. We found that when a key meningococcal surface component was deleted, several interactions previously seen with the wild-type strain were lost. These results suggest that the interactions may be mediated by the mutated surface components.

Comparison of wild-type MC58 with the non-encapsulated mutant revealed both loss and gain of interactions on the glycan array. The additional binding revealed when capsule was removed was expected, as the ability of capsule to supress meningococcal host pathogen interactions is well known and is thought to be due to the presence of this dense high molecular weight polyanionic polysaccharide coating the bacterial surface^32, 33^. The capsule’s masking effects include inefficient Opc mediated invasion in encapsulated meningococci^5^. Consequently, the interactions lost with the pili mutants, varied depending on the presence/absence of the capsule. As the capsule is crucial for meningococcal survival in serum/CSF^1^ and the non-encapsulated state is favoured during colonisation, the variation in glycan binding by the different pili mutants, suggests a flexibility in the roles that pili play in these interactions, depending on disease stage. Interestingly, the non-encapsulated pili mutant lost binding to asialo GM1 (_IN_382) and GalNAcβ1-4 Gal (_IN_2D) (Supplementary Table 1), implying that meningococcal pili mediate these interactions. Asialo GM1 is highly expressed in regenerating respiratory epithelia and is a potential target for meningococcal adherence to these cells. Several pathogens that colonise the respiratory tract, are known to target asialo GM1 (or -GM2)^34^ by binding specifically to its sub-terminal GalNAcβ1-4 Gal epitope. For *Pseudomonas aeruginosa*, this interaction is also mediated by pili^35^. Moreover, a similar study comparing glycan binding of a wild type *Vibrio parahaemolyticus* strain to a mutant lacking the mannose-sensitive haemagglutinin (MSHA) pilus, showed a loss of binding to asialo GM1 ganglioside by the mutant^29^. Hence the ability of meningococci to bind asialo GM1 and its sub-terminal structure implies a similar mechanism may be used by these organisms.

Interestingly, our findings show that the capsule may also mediate binding to glycans as binding to 43 structures was lost in the capsule mutant. Recent evidence suggests that high affinity glycan-glycan interactions are a common mechanism of bacterial adherence to host glycans, including by high molecular weight lipopolysaccharides^15^. A detailed study of the structural basis for the interactions between the (α2-8)-linked polysialic acid capsule and host glycan structures is the subject of our current investigations.

Glycan array analysis of wild-type and mutant strains allowed us to putatively ascribe numerous interactions to Opc protein. Opc has well documented adherence and invasion functions^5, 13, 16^, and has been shown to bind a range of glycans/glycoconjugates^16^. In this study, we have revealed novel glycan binding by Opc. We show high affinity Opc binding to chondroitin-6-sulfate polymer and lacto-*N*-neotetraose. The chondroitin-6-sulfate polymer is found in all human extra cellular matrix, and is the major GAG expressed within the CNS^24^, suggesting the potential for Opc-mediated meningococcal adherence to cells within the CNS during meningitis^4^. Lacto-*N*-neotetraose is an integral component of lacto-neo series glycosphingolipids such as paragloboside^36^, and is also the precursor of the ABO and P1 blood group antigens^37^. These glycosphingolipids and blood group antigens are expressed ubiquitously on host cells^38, 39^ and are abundant on neuronal^40^ and red blood cells^41^, respectively. ABO Blood group antigens are also among the most common terminal glycans found on mucins^42^ and may be important for naso-pharyngeal colonization. Lacto-*N*-neotetraose structures on nasopharyngeal epithelia are known to mediate adherence of other bacteria such as *Streptococcus pneumoniae*. Studies have shown that free lacto-*N*-neotetraose inhibits pneumococcal adherence to human^43^ and animal^44^ nasopharyngeal cells.

Natural LOS phase variants (L3 and L8) were used for the investigation of LOS mediated glycointeractions. Results showed that the variants bind different glycans, and this suggests a variation in the roles of meningococcal immunotypes with respect to host pathogen interactions. Interestingly, glycan array analysis of purified LOS showed conflicting results to that seen for the whole bacteria. In that regard, the fact that the purified L3 LOS recognised more glycans than the purified L8, implies that the L3 LOS negatively modulates the meningococcal interactions attributed to other meningococcal surface components, such as pili or Opc. These findings substantiate earlier studies where sialylated LOS was shown to interfere with Opc - host glycan interactions^13^. LOS of both the L3 and L8 immunotype structures bound α2-8 linked sialic acid saccharides ((sia)_3_ and/or colominic acid). These α2-8 linked sialic acid polymers are abundantly expressed in the human neuronal tissues where they are found associated with the neural cell adhesion molecule (NCAM)^23^.

The highest affinity interaction for LOS was observed between L3 LOS and the TF antigen. This interaction is the highest affinity glycan-glycan interaction described to date^15^. TF antigen is the most common mucin O-glycan core (core 1) structure which is found on most cell types^14^. Interestingly, binding to TF antigen was observed with L3 LOS but not with L8 LOS immunotypes, indicating that the terminal lacto-*N*-neotetraose epitope present only in L3 (Fig. 2), is required for the binding to TF antigen.

The human glycome is extensive and is an important target for interactions with bacterial pathogens. We have shown that *N. meningitidis* MC58 binds glycans from different structural and functional classes. Some of these interactions are lost when key outer membrane components are deleted, suggesting that these components may mediate glycan binding. The meningococcal surface factors responsible for the novel glycan interactions described herein may be the basis for new meningococcal-host interactions and may provide new strategies to prevent and treat meningococcal disease.

## Materials and Methods

### Bacterial strains and growth conditions

*N. meningitidis* MC58 wild-type, mutant strains and phase variants used in this study (Table 1) were grown on BHI supplemented with Leventhal’s base and incubated overnight at 37 °C at 5% CO_2._

MC58 and Ȼ3 pili knockout mutants (MC58Δ*pilE* and Ȼ3Δ*pilE*, respectively) were generated using a PCR product from the *N. meningitidis* C311*#3*Δ*pilE* mutant that has a kanamycin resistance cassette in *pilE* ^45^. Primers *pilE*For (5′-GCCGTCTGAAATGAACACCCTTCAA AAAGGTTTTACCCTT-3′) and *pilE*Rev (5′-TTCAGACGGCATAAACCGCTTCCTTATC AAGGGGGTAAGT-3′) were used and the PCR product was purified using the QIAGEN PCR purification kit, and transformed into MC58 and Ȼ3 as previously described^45^.

In order to construct an *lst::kan* knockout mutant, a kanamycin antibiotic resistance cassette was inserted into the *lst* coding sequence. This involved the introduction of a unique BamHI restriction site into the *lst* gene by PCR. Oligonucleotide primers S3 5′-CG**GGATCC**GCGGGCGTACTTTTTCCA-3′ (BamHI site in bold) and S5 5′-TGCCGTC TGAAGACTTCAGACGGCTATCGTCAAATGTCAAAAT-3′ (uptake sequence underlined) were used to amplify the 3′-end of the *lst* gene and the resulting PCR product cloned into pT7Blue (Novogen) to create pST3. Primers S1 5′-CG**GAATTC**GTCGGT ATGGGTATAAACA-3′ (EcoRI site in bold) and S2 5′-CG**GGATCC**TTATCCTTTAT CTGATTG-3′ (BamHI site in bold) were used to amplify the 5′-end of the *lst* gene. The resulting PCR product was digested with BamHI/EcoRI and ligated, along with the BamHI fragment from pUC4kan (Pharmacia), to BamHI/EcoRI cut pST3, to create pSTkan8. This plasmid was used transformed into Ȼ3 as previously described^45^. The Ȼ3Δ*lst* mutant was selected on BHI agar with 100 µg/ml kanamycin, and confirmed by PCR using primers S3 and S5.

### LOS purification

LOS was purified from *N. meningitidis* wild-type, Ȼ3, Ȼ3Δ*lst* and Ȼ3Δ*lgtABE* strains as previously described^46^. Supplementary Figure S1 shows LOS purified from these strains. Briefly, bacteria were grown as described above, harvested into phosphate buffered saline pH 7.4 (PBS; Sigma), pelleted (5000 × g, 4 °C, 15 min) then lyophilised. The LOS was extracted from the dried biomass using hot phenol extraction^46^. Purified LOS was lyophilised and weighed for quantification. LOS was hydrolysed by heating in 1% (vol/vol) acetic acid as previously described^15^.

### Glycan array analysis of bacteria and purified LOS

Glycan array analysis was performed as previously described by Day *et al* ^15^ using array v3.0 from the Institute of Glycomics^47^. Briefly, bacteria were harvested into PBS and fixed with 2.5% formaldehyde (30 min), labelled with 10 μM Bodipy558-succinimidyl ester (30 min), and washed three times in PBS. Cells were pelleted (771 × *g*, for 5 min), resuspended in PBS + 2 mM MgCl_2_, 2 mM CaCl_2_ (array PBS) and adjusted to OD 600 nm = 0.1. All buffers were filtered prior to use. Labelled bacteria (125 μL; labelling confirmed by flow cytometry^48^ were added to the array slide and hybridized for 30 min. Purified LOS (1 µg) was labelled with lipophilic Bodipy methyl ester 595/625 cell trace, and 125 μL was added to the array slide and hybridized for 30 min, as previously described^15^. After incubation, slides were washed three times with array PBS, dried by centrifugation (200 × *g*, 4 min), scanned using a ProScan Array scanner, and the results analysed using the ScanArray Express software program. Positive binding is defined as binding to all four replicate spots on each array, in all three replicate experiments. Binding for each spot was defined as a value greater than 1-fold above the mean background relative fluorescence units (RFU). The mean background was calculated from the average background of empty spots on the array plus three standard deviations. Statistical analysis of the data was performed by a Student’s *t*-test with a confidence level of 99.99% (*p* ≤ 0.0001).

### Isothermal calorimetry (ITC) analysis of Opc - host glycan interactions

ITC analysis was performed as previously described^15^ using outer membrane preparations (OMPs) with/without Opc which were extracted from *N. meningitidis* strains Ȼ4 (Opc^+^) and Ȼ11 (Opc^−^), respectively. As opposed to Ȼ2 (Opc^+^) and Ȼ9 (Opc^−^) which were used for whole cells assays, Ȼ4 and Ȼ11 express fewer outer proteins (Table 1) and thus allowed for the extraction of relatively pure OMP preps (Supplementary Fig. S2).

Bacterial strains were grown to mid log-phase (4 hours) and OMPs were prepared using sarkosyl as previously described^49^. OMPs were resuspended in PBS and quantified using the Pierce BCA Protein Assay Kit (Thermo Fisher Scientific). ITC was performed using a nano-ITC (TA instruments) with 170 µl (4 mg/ml) of Opc+ OMPs in the calorimeter cell and 50 µl (100 µM) glycan in the syringe. A fixed OMP concentration was used in the ITC cell, due to the large and complex thermodynamic changes of injecting lipid and detergent containing solution such as the OMPs at increasing concentrations^50^, with glycan titrated into the sample. A total of 20 injections of 2.5 µL glycan were added sequentially at 5 min intervals. Opc− OMPs-glycan and PBS-glycan interactions were run as negative controls. The Opc− OMPs - glycan controls were performed as described above for Opc + , and the data collected was background subtracted from the Opc+ - glycan interaction with injection per injection subtraction. For the PBS control, 50 µL (100 µM) glycan was injected in 20 injections of 2.5 µL into PBS (170 µl) only, providing a measure (single value) of heat caused by the injection of glycan into a liquid. This was also background subtracted. The glycan asialo GM1 was also used as a negative control, based on binding of both the wild type and Opc- mutant strains on the glycan array. Affinity data was determined for a minimum of two repeats and the average affinity constants (K_D_ values) obtained are reported.

### Surface plasmon resonance (SPR) analysis of LOS - host glycan interactions

SPR was performed using the Biacore T100 system and Series S L1 sensor chip as described previously^15^. Briefly, LOS extracted from *N. meningitidis* strains MC58, Ȼ3, Ȼ3Δ*lst* and Ȼ3Δlgt*ABE* (as described above) was captured onto an L1 Chip at 100 µg/ml. The unglycosylated lipid A obtained from hydrolysed Ȼ3ΔlgtABE LOS, Ȼ3Δ*lgtABE* was used as the negative control on flow cell 1, as described previously^51^. Initial screening was done with a 1:5 dilution series from 0.1 to 10 µM. All SPR experiments were repeated at least three times.

## Electronic supplementary material

Supplementary information
