# Supplementary material for: The glycointeractome of serogroup B Neisseria meningitidis strain MC58
Source: Sci Rep. 2017 Jul 18;7:5693. doi: 10.1038/s41598-017-05894-w (PMC5515891; doi:10.1038/s41598-017-05894-w)

The glycointeractome of serogroup B *Neisseria meningitidis* strain MC58

Tsitsi D. Mubaiwa, Lauren E. Hartley-Tassell, Evgeny A. Semchenko, Freda. E.-C. Jen, Yogitha N. Srikhanta[#], Christopher J. Day, Michael P. Jennings*, Kate L. Seib*

Institute for Glycomics, Griffith University, Gold Coast, Queensland, Australia

* Address correspondence to m.jennings@griffith.edu.au, k.seib@griffith.edu.au

Supplementary material includes:

Table S1: Glycan array results of *Neisseria meningitidis* MC58 whole cells and purified lipooligosaccharide (LOS).

Figure S1. Silver-stained Tricine-SDS-PAGE gel showing LOS purified from *N. meningitidis* Ȼ3, Ȼ3*Δlst*, Ȼ2 and Ȼ3*ΔlgtABE* strains.

Figure S2. Coomassie Blue stained Bis-Tris SDS-PAGE gel showing outer membrane preparations from the Opc expressing strain (Ȼ4) and the isogenic Opc mutant strain (Ȼ11).

Dataset S1. Representative (i) sensograms and (ii) sigmoidal curves from isothermal calorimetry (ITC) analysis of Opc interactions.

Dataset S2. Representative sensograms from surface plasmon resonance (SPR) analysis of lipooligosaccharide (LOS)-glycan interactions.

Supplementary Table S1: Glycan array results of *Neisseria meningitidis* MC58 whole cells and purified lipooligosaccharide (LOS).

Glycan			Strain						Purified LOS		
**Class**	**Index**	**Structure**	**MC58**	**MC58Δ*pilE***	**Ø3**	**Ø3Δ*pilE***	**Ø2**	**Ø9**	**L3**	**L8**	**Δ*lgtABE***
Terminal Gal	2	Galα-sp3									
	3	Galβ-sp3		■	■	■	■	■			
	37	3-O-Su-Galβ-sp3					■				
	75	Galα1-2Galβ-sp3	■	■	■	■	■	■			
	76	Galα1-3Galβ-sp3	■		■		■				
	77	Galα1-3GalNAcβ-sp3				■	■	■			
	78	Galα1-3GalNAcα-sp3	■	■	■	■	■	■			
	80	Galα1-3GlcNAcβ-sp3		■	■	■	■	■			
	81	Galα1-4GlcNAcβ-sp3	■	■	■	■	■				
	83	Galα1-6Glcβ-sp4		■	■	■	■	■			
	84	Galβ1-2Galβ-sp3	■			■	■	■			
	85	Galβ1-3GlcNaAcβ-sp3				■	■	■			
	87	Galβ1-3Galβ-sp3									
	88	Galβ1-3GalNAcβ-sp3	■		■	■	■	■			
	89	Galβ1-3GalNAcα-sp3			■				■		
	93	Galβ1-4Glcβ-sp4		■	■	■	■				
	94	Galβ1-4Galβ-sp4			■	■					
	97	Galβ1-4GlcNAcβ-sp3				■					
	100	Galβ1-6Galβ-sp4									
	145	Galβ1-3(6-O-Su)GlcNAcβ-sp3			■	■	■	■			
	146	Galβ1-4(6-O-Su)Glcβ-sp2	■		■	■	■				
	147	Galβ1-4(6-O-Su)GlcNAcβ-sp3	■	■	■	■	■	■			
	150	3-O-Su-Galβ1-3GalNAcα-sp3	■		■	■	■	■			
	151	6-O-Su-Galβ1-3GalNAcα-sp3			■	■	■	■			
	152	3-O-Su-Galβ1-4Glcβ-sp2		■	■	■	■		■		
	153	6-O-Su-Galβ1-4Glcβ-sp2	■	■	■	■	■	■			
	155	3-O-Su-Galβ1-3GlcNAcβ-sp3									
	157	3-O-Su-Galβ1-4GlcNAcβ-sp3	■			■	■	■			
	159	4-O-Su-Galβ1-4GlcNAcβ-sp3	■	■	■	■	■	■			
	161	6-O-Su-Galβ1-3GlcNAcβ-sp3	■	■	■		■	■			
	163	6-O-Su-Galβ1-4GlcNAcβ-sp3			■		■				
	176	3-O-Su-Galβ1-4(6-O-Su)Glcβ-sp2	■	■	■	■	■	■	■		
	177	3-O-Su-Galβ1-4(6-O-Su)GlcNAcβ-sp2	■	■	■	■	■	■			
	178	6-O-Su-Galβ1-4(6-O-Su)Glcβ-sp2	■	■	■	■	■	■			
	179	6-O-Su-Galβ1-3(6-O-Su)GlcNAcβ-sp2	■	■	■	■	■	■			

180	6-O-Su-Galβ1-4(6-O-Su)GlcNAcβ-sp2
181	3,4-O-Su$_2$-Galβ1-4GlcNAcβ-sp3
182	3,6-O-Su$_2$-Galβ1-4GlcNAcβ-sp2
183	4,6-O-Su$_2$-Galβ1-4GlcNAcβ-sp2
184	4,6-O-Su$_2$-Galβ1-4GlcNAcβ-sp3
189	3,6-O-Su$_2$-Galβ1-4(6-O-Su)GlcNAcβ-sp2
201	3,4-O-Su$_2$-Galβ1-4GlcNAcβ-sp3
203	Galβ1-4(6-O-Su)GlcNAcβ-sp2
220	Galα1-3Galβ1-4Glcβ-sp2
222	Galα1-3Galβ1-4GlcNAcβ-sp3
224	Galα1-4Galβ1-4Glcβ-sp3
225	Galα1-4Galβ1-4GlcNAc-sp2
228	Galβ1-2Galα1-4GlcNAcβ-sp4
229	Galβ1-3Galβ1-4GlcNAcβ-sp4
231	Galβ1-4GlcNAcβ1-3GalNAcα-sp3
232	Galβ1-4GlcNAcβ1-6GalNAcα-sp3
254	Galβ1-3(GlcNAcβ1-6)GalNAcα-sp3
262	Galβ1-3GalNAcβ1-3Gal-sp4
264	Galβ1-4Galβ1-4GlcNAc-sp3
373	Galα1-3Galβ1-4GlcNAcβ1-3Galβ-sp3
375	Galα1-4GlcNAcβ1-3Galβ1-4GlcNAcβ-sp3
376	Galβ1-3GlcNAcβ1-3Galβ1-4Glcβ-sp4
377	Galβ1-3GlcNAcβ1-3Galβ1-3GlcNAcβ-sp2
378	Galβ1-3GlcNAcα1-3Galβ1-4GlcNAcβ-sp3
379	Galβ1-3GlcNAcβ1-3Galβ1-4GlcNAcβ-sp3
380	Galβ1-3GlcNAcα1-6Galβ1-4GlcNAcβ-sp2
381	Galβ1-3GlcNAcβ1-6Galβ1-4GlcNAcβ-sp2
382	Galβ1-3GalNAcβ1-4Galβ1-4Glcβ-sp3
383	Galβ1-4GlcNAcβ1-3Galβ1-4Glcβ-sp2
385	Galβ1-4GlcNAcβ1-3Galβ1-4GlcNAcβ-sp3
387	Galβ1-4GlcNAcβ1-6Galβ1-4GlcNAcβ-sp2
388	Galβ1-3(Galβ1-4GlcNAcβ1-6)GalNAcα-sp3
401	Galβ1-3GlcNAcβ1-3Galβ1-3GlcNAcβ-sp3
419	3-O-SuGalβ1-4GlcNAcβ1-3Galβ1-4GlcNAcβ-sp3
420	4-O-SuGalβ1-4GlcNAcβ1-3Galβ1-4GlcNAcβ-sp3
481	Galα1-3Galβ1-4GlcNAcβ1-3Galβ1-4Glcβ-sp4
488	Galβ1-4GlcNAcβ1-3(Galβ1-4GlcNAcβ1-6)GalNAcα-sp3
489	Galβ1-4GlcNAcβ1-3(GlcNAcβ1-6)Galβ1-4GlcNAc-sp2
490	Galβ1-4GlcNAcβ1-6(GlcNAcβ1-3)Galβ1-4GlcNAcβ-sp2
498	(Galβ1-4GlcNAcβ1-3)$_3$-sp3
499	Galβ1-4GlcNAcβ1-3(Galβ1-4GlcNAcβ1-6)Galβ1-4GlcNAc-sp2
501	Galβ1-3GalNAcβ1-3Galα1-4Galβ1-4Glcβ-sp4
1A	Galβ1-3GlcNAc
1B	Galβ1-4GlcNAc

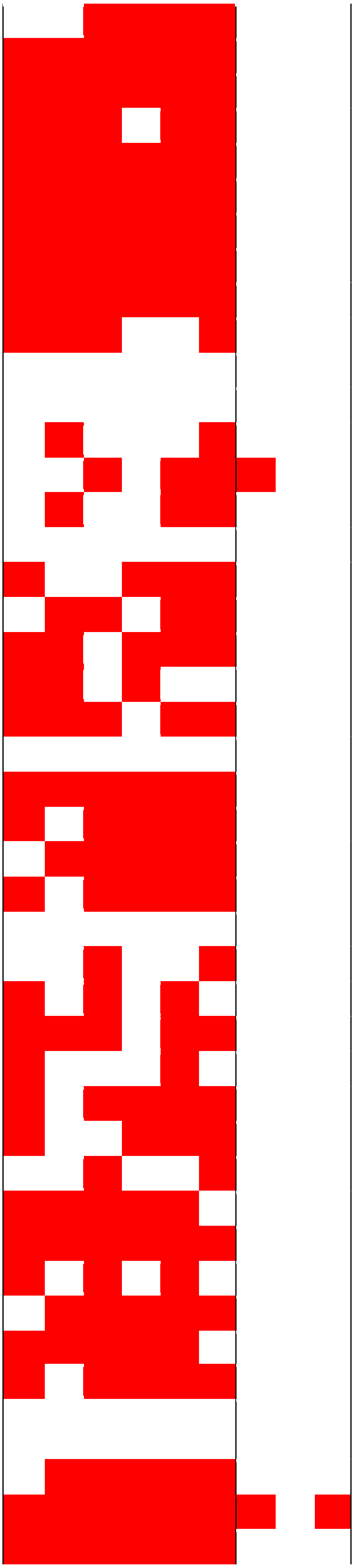

	1C	Galβ1-4Gal
	1D	Galβ1-6GlcNAc
	1E	Galβ1-3GalNAc
	1F	Galβ1-3GalNAcβ1-4Galβ1-4Glc
	1G	Galβ1-3GlcNAcβ1-3Galβ1-4Glc
	1H	Galβ1-4GlcNAcβ1-3Galβ1-4Glc
	1I	Galβ1-4GlcNAcβ1-6(Galβ1-4GlcNAcβ1-3)Galβ1-4Glc
	1J	Galβ1-4GlcNAcβ1-6(Galβ1-3GlcNAcβ1-3)Galβ1-4Glc
	1K	Galα1-4Galβ1-4Glc
	1L	GalNAcα1-O-Ser
	1M	Galβ1-3GalNAcα1-O-Ser
	1N	Galα1-3Gal
	1O	Galα1-3Galβ1-4GlcNAc
	1P	Galα1-3Galβ1-4Glc
	2A	Galα1-3Galβ1-4Galα1-3Gal
	2B	Galβ1-6Gal
	2C	GalNAcβ1-3Gal
	2D	GalNAcβ1-4Gal
	2E	Galα1-4Galβ1-4GlcNAc
	2F	GalNAcα1-3Galβ1-4Glc
	2G	Galβ1-3GlcNAcβ1-3Galβ1-4GlcNAcβ1-6(Galβ1-3GlcNAcβ1-3)Galβ1-4Glc
**Terminal GlcNAc**	10	GlcNAcβ-sp3
	22	GlcNAcβ-sp4
	43	6-O-Su-GlcNAcβ-sp3
	55	3-O-Su-GlcNAcβ-sp3
	113	GlcNAcβ1-3GalNAcα-sp3
	114	GlcNAcβ1-3Manβ-sp4
	115	GlcNAcβ1-4GlcNAcβ-Asn
	117	GlcNAcβ1-4GlcNAcβ-sp4
	118	GlcNAcβ1-6GalNAcα-sp3
	149	GlcNAcβ1-4(6-O-Su)GlcNAcβ-sp2
	167	GlcNAcβ1-4-[HOOC(CH_3)CH]-3-O-GlcNAcβ-sp4
	168	GlcNAcβ1--[HOOC(CH_3)CH]-3-O-GlcNAcβ-L-alanyl-D-i-glutaminyl-L-lysine
	246	GlcNAcβ1-2Galβ1-3GalNAcα-sp3
	247	GlcNAcβ1-3Galβ1-3GalNAcα-sp3
	248	GlcNAcβ1-3Galβ1-4Glcβ-sp2
	250	GlcNAcβ1-3Galβ1-4GlcNAcβ-sp3
	251	GlcNAcβ1-4Galβ1-4GlcNAcβ-sp2
	252	GlcNAcβ1-4GlcNAcβ1-4GlcNAcβ-sp4
	253	GlcNAcβ1-6Galβ1-4GlcNAcβ-sp2
	255	GlcNAcβ1-3(GlcNAcβ1-6)GalNAcα-sp3
	395	GlcNAcβ1-3(GlcNAcβ1-6)Galβ1-4GlcNAcβ-sp3
	493	$(GlcNAcβ1\text{-}4)_5$β-sp4
	503	$(GlcNAcβ1\text{-}4)_6$β-sp4

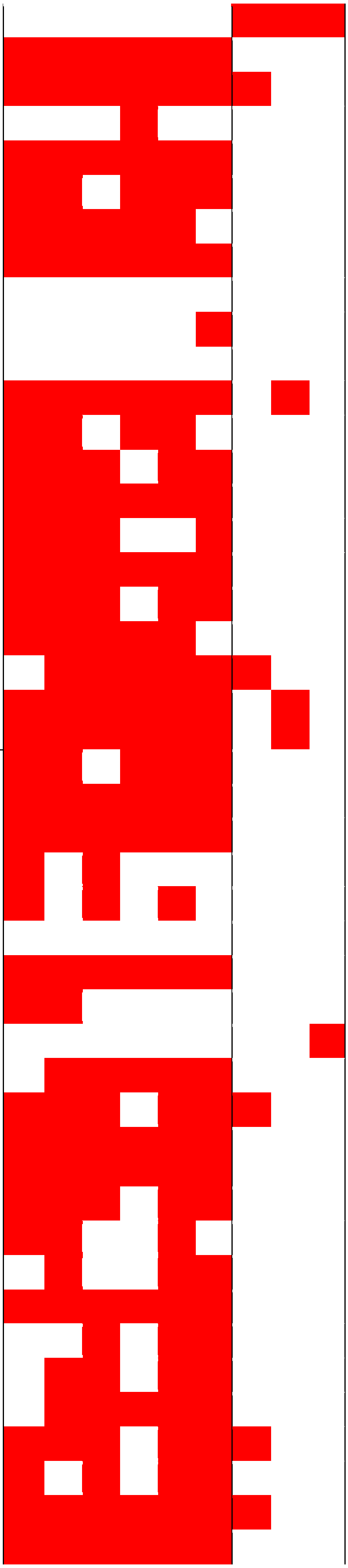

	504	(A-GN-M)$_2$-3,6-M-GN-GNβ-sp4
	505	(GN-M)$_2$-3,6-M-GN-GNβ-sp4
	4A	GlcNAcβ1-4GlcNAc
	4B	GlcNAcβ1-4GlcNAcβ1-4GlcNAc
	4C	GlcNAcβ1-4GlcNAcβ1-4GlcNAcβ1-4GlcNAc
	4D	GlcNAcβ1-4GlcNAcβ1-4GlcNAcβ1-4GlcNAcβ1-4GlcNAcβ1-4GlcNAc
	4E	GlcNAcβ1-4MurNAc
**Mannosyl**	16	Manα-sp3
	18	Manβ-sp4
	19	ManNAcβ-sp4
	47	6-H_2PO_3Manα-sp3
	119	Manα1-2Manβ-sp4
	120	Manα1-3Manβ-sp4
	121	Manα1-4Manβ-sp4
	122	Manα1-6Manβ-sp4
	123	Manβ1-4GlcNAcβ-sp4
	124	Manα1-2Manα-sp4
	258	Manα1-3(Manα1-6)Manβ-sp4
	495	Manα1-6(Manα1-3)Manα1-6(Manα1-3) Manβ-sp4
	5A	GlcNAcβ1-2Man
	5B	GlcNAcβ1-2Manα1-6(GlcNAcβ1-2Manα1-3)Man
	5C	Manα1-2Man
	5D	Manα1-3Man
	5E	Manα1-4Man
	5F	Manα1-6Man
	5G	Manα1-6(Manα1-3)Man
	5H	Manα1-6(Manα1-3)Manα1-6(Manα1-3) Man
**Fucosyl**	1	Fucα-sp3
	71	Fucα1-2Galβ-sp3
	72	Fucα1-3GlcNAcβ-sp3
	73	Fucα1-4GlcNAcβ-sp3
	215	Fucα1-2Galβ1-3GlcNAcβ-sp3
	216	Fucα1-2Galβ1-4GlcNAcβ-sp3
	217	Fucα1-2Galβ1-3GalNAcα-sp3
	219	Fucα1-2Galβ1-4Glcβ-sp4
	226	Fucα1-2(Galα1-3)Galβ-sp3
	233	Galβ1-3(Fucα1-4)GlcNAcβ-sp3
	234	Fucα1-3(Galβ1-4)GlcNAcβ-sp3
	235	Fucα1-2(GalNAcα1-3)Galβ-sp3
	287	3-O-Su-Galβ1-3(Fucα1-4)GlcNAcβ-sp3
	288	Fucα1-3(3-O-Su-Galβ1-4)GlcNAcβ-sp3
	359	Fucα1-2(Galα1-3)Galβ1-3GlcNAcβ-sp3
	360	Fucα1-2(Galα1-3)Galβ1-4GlcNAcβ-sp3
	362	Fucα1-2(Galα1-3)Galβ1-3GalNAcα-sp3

363	Fucα1-2(Galα1-3)Galβ1-3GalNAcβ-sp3	
364	Fucα1-3(Galα1-3Galβ1-4)GlcNAcβ-sp3	
366	Fucα1-2(GalNAcα1-3)Galβ1-3GlcNAcβ-sp3	
368	Fucα1-2(GalNAcα1-3)Galβ1-4GlcNAcβ-sp3	
371	Fucα1-2Galβ1-3(Fucα1-4)GlcNAcβ-sp3	
372	Fucα1-3(Fucα1-2Galβ1-4)GlcNAcβ-sp3	
392	Fucα1-2(GalNAcα1-6)GalNAcα1-6-sp3	
479	Fucα1-2Galβ1-3GlcNAcβ1-3Galβ1-4Glcβ-sp4	
480	Fucα1-2Galβ1-3GlcNAcβ1-3Galβ1-4GlcNAcβ-sp2	
483	Galα1-3(Fucα1-2)Galβ1-4 (Fucα1-3)GlcNAcβ-sp3	
496	Fucα1-2Galβ1-3(Fucα1-4)GlcNAcβ1-3Galβ1-4Glcβ-sp4	
497	Fucα1-3(Fucα1-2Galβ1-4)GlcNAcβ1-3Galβ1-4Glcβ-sp4	
538	Galβ1-4(Fucα1-3)GlcNAcβ1-6(Galβ1-3GlcNAc1-3)Galβ1-4Glcβ-sp4	
539	Galβ1-4GlcNAcβ1-6(Fucα1-2Galβ1-3GlcNAcβ1-3)Galβ1-4Glcβ-sp4	
540	Galβ1-4(Fucα1-3)GlcNAcβ1-6 (Neu5Acα2-6Galβ1-4GlcNAcβ1-3)Galβ1-4Glcβ-sp4	
541	Galβ1-4(Fucα1-3)GlcNAcβ1-6 (Fucα1-2Galβ1-3GlcNAcβ1-3)Galβ1-4Glcβ-sp4	
542	Galβ1-3GlcNAcβ1-3Galβ1-4(Fucα1-3)GlcNAcβ1-6 (Galβ1-3GlcNaAcβ1-3 Galβ1-4Glcβ-sp4	
543	Galβ1-4(Fucα1-3)GlcNAcβ1-6 (Fucα1-2Galβ1-3(Fucα1-4)GlcNAcβ1-3) Galβ1-4Glcβ-sp4	
7A	Fucα1-2Galβ1-3GlcNAcβ1-3Galβ1-4Glc	
7B	Galβ1-3(Fucα1-4)GlcNAcβ1-3Galβ1-4Glc	
7C	Galβ1-4(Fucα1-3)GlcNAcβ1-3Galβ1-4Glc	
7D	Fucα1-2Galβ1-3(Fucα1-4)GlcNAcβ1-3Galβ1-4Glc	
7E	Galβ1-3(Fucα1-4)GlcNAcβ1-3Galβ1-4(Fucα1-3)Glc	
7F	Fucα1-2Gal	
7G	Fucα1-2Galβ1-4Glc	
7H	Galβ1-4(Fucα1-3)Glc	
7I	Galβ1-4(Fucα1-3)GlcNAc	
7J	Galβ1-3(Fucα1-4)GlcNAc	
7K	GalNAcα1-3(Fucα1-2)Gal	
7L	Fucα1-2Galβ1-4(Fucα1-3)Glc	
7M	Galβ1-3(Fucα1-2)Gal	
7N	Fucα1-2Galβ1-4(Fucα1-3)GlcNAc	
7O	Fucα1-2Galβ1-3GlcNAc	
7P	Fucα1-2Galβ1-3(Fucα1-4)GlcNAc	
8A	SO_3-3Galβ1-3(Fucα1-4)GlcNAc	
8B	SO_3-3Galβ1-4(Fucα1-3)GlcNAc	
8C	Galβ1-3GlcNAcβ1-3Galβ1-4(Fucα1-3)GlcNAcβ1-3Galβ1-4Glc	
8D	Galβ1-4(Fucα1-3)GlcNAcβ1-6(Galβ1-3GlcNAcβ1-3)Galβ1-4Glc	

	8E	Galβ1-4(Fucα1-3)GlcNAcβ1-6(Fucα1-2Galβ1-3GlcNAcβ1-3)Galβ1-4Glc
	8F	Galβ1-4(Fucα1-3)GlcNAcβ1-6(Fucα1-2Galβ1-3(Fucα1-4)GlcNAcβ1-3)Galβ1-4Glc
	8G	Galβ1-4GlcNAcβ1-3Galβ1-4(Fucα1-3)Glc
	8H	Fucα1-2Galβ1-4(Fucα1-3)GlcNAcβ1-3Galβ1-4Glc
	8I	Fucα1-3Galβ1-4GlcNAcβ1-3Galβ1-4(Fucα1-3)Glc
	8J	Fucα1-2Galb1-4(Fucα1-3)GlcNAcb1-3(Fucα1-2)Galb1-4Glc
	8K	Galβ1-4(Fucα1-3)GlcNAcβ1-6(Galβ1-4GlcNAcβ1-3)Galβ1-4Glc
	8L	Galβ1-4(Fucα1-3)GlcNAcβ1-6(Galβ1-4(Fucα1-3)GlcNAcβ1-3)Galβ1-4Glc
	8M	Fucα1-2Galβ1-4(Fucα1-3)GlcNAcβ1-6(Galβ1-4GlcNAcβb1-3)Galβ1-4Glc
	8N	Galβ1-3GlcNAcβ1-3Galβ1-4(Fucα1-3)GlcNAcβ1-6(Galβ1-3GlcNAcβ1-3)Galβ1-4Glc
	8O	Fucα1-2Galβ1-3GlcNAcβ1-3Galβb1-4(Fucα1-3)GlcNAcβ1-6(Galβ1-3GlcNAcβ1-3)Galβ1-4Glc
**Sialylated**	48	Neu5Acα-sp3
	49	Neu5Acα-sp9
	52	Neu5Gcα-sp3
	54	9-NAc-Neu5Acα-sp3
	169	Neu5Acα2-3Galβ-sp3
	170	Neu5Acα2-6Galβ-sp3
	171	Neu5Acα2-3GalNAcα-sp3
	172	Neu5Acα2-6GalNAcα-sp3
	174	Neu5Gcα2-6GalNAcα-sp3
	186	Neu5Acα2-8Neu5Acα2-sp3
	205	Neu5Acα2-6GalNAcβ-sp3
	206	Neu5Gcα2-3Gal-sp3
	289	Galα1-3(Neu5Acα2-6)GalNAcα-sp3
	290	Galβ1-3(Neu5Acα2-6)GalNAcα-sp3
	292	Neu5Acα2-3Galβ1-3GalNAcα-sp3
	293	Neu5Acα2-3Galβ1-4Glcβ-sp3
	294	Neu5Acα2-3Galβ1-4Glcβ-sp4
	295	Neu5Acα2-6Galβ1-4Glcβ-sp2
	298	Neu5Acα2-3Galβ1-4GlcNAcβ-sp3
	299	Neu5Acα2-3Galβ1-3GlcNAcβ-sp3
	300	Neu5Acα2-6Galβ1-4GlcNAcβ-sp3
	303	Neu5Gcα2-3Galβ1-4GlcNAcβ-sp3
	304	Neu5Gcα2-6Galβ1-4GlcNAcβ-sp3
	306	9-NAc-Neu5Acα2-6Galβ1-4GlcNAcβ-sp3
	315	Neu5Acα2-3Galβ1-4-(6-O-Su)GlcNAcβ-sp3
	317	Neu5Acα2-3Galβ1-3-(6-O-Su)GalNAcβ-sp3
	318	Neu5Acα2-6Galβ1-4-(6-O-Su)GlcNAcβ-sp3
	319	Neu5Acα2-3-(6-O-Su)Galβ1-4GlcNAcβ-sp3
	321	(Neu5Acα2-8)$_3$-sp3

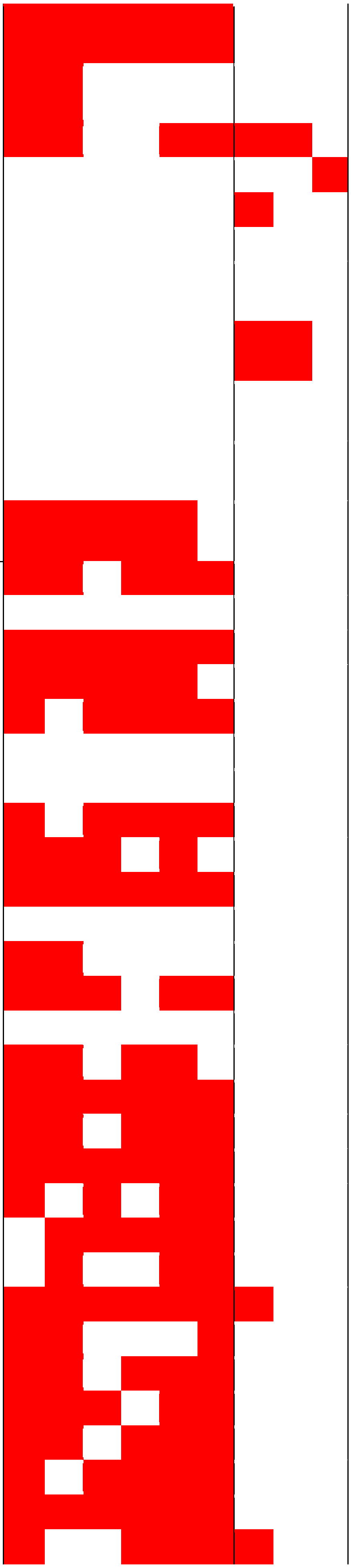

	323	Neu5Acα2-6Galβ1-3GlcNAc-sp3
	324	Neu5Acα2-6Galβ1-3(6-O-Su)GlcNAc-sp3
	331	Neu5Gcα2-3Galβ1-3GlcNAcβ-sp3
	421	Neu5Acα2-3(GalNAcβ1-4)Galβ1-4Glcβ-sp2
	422	Neu5Acα2-3Galβ1-4GlcNAcβ1-3Galβ-sp3
	423	Fucα1-3(Neu5Acα2-3Galβ1-4)GlcNAcβ-sp3
	426	Neu5Acα2-3Galβ1-3(Fucα1-4)GlcNAcβ-sp3
	428	Fucα1-3(Neu5Acα2-3Galβ1-4)6-O-Su-GlcNAcβ-sp3
	429	Fucα1-3(Neu5Acα2-3(6-O-Su)Galβ1-4)GlcNAcβ-sp3
	433	Neu5Acα2-3Galβ1-3(Neu5Acα2-6)GalNAcα-sp3
	434	Neu5Acα2-8Neu5Acα2-3Galβ1-4Glcβ-sp4
	527	Neu5Acα2-3Galβ1-4GlcNAcβ1-3Galβ1-4GlcNAcβ-sp2
	528	Fucα1-3(Neu5Acα2-3Galβ1-4)GlcNAcβ1-3Galβ-sp3
	529	Galβ1-3(Neu5Acα2-6)GlcNAcβ1-3Galβ1-4Glcβ-sp4
	531	Neu5Acα2-8Neu5Acα2-3(GalNAcβ1-4)Galβ1-4Glc-sp2
	532	Neu5Acα2-8Neu5Acα2-8Neu5Acα2-3Galβ1-4Glc-sp2
	533	(Neu5Acα2-8)2Neu5Acα2-3(GalNAcβ1-4)Galβ1-4Glc-sp2
	534	Neu5Acα2-3Galβ1-4GlcNAcβ1-3Galβ1-4GlcNAcβ-sp3
	536	Neu5Acα2-3Galβ1-3GlcNAcβ1-3Galβ1-4Glcβ-sp4
	537	Neu5Acα2-3Galβ1-4GlcNAcβ1-3Galβ1-4Glcβ-sp4
	540	Fucα1-3(Galβ1-4)GlcNAcβ1-6(Neu5Acα2-3Galβ1-4GlcNAcβ1-3)Galβ1-4Glcβ-sp4
	627	Neu5Acα2-6Galβ1-3GlcNAcβ1-4Manα1-6(Neu5Acα2-6Galβ1-3GlcNAcβ1-4Manα1-3)Manβ1-4GlcNAcβ1-4GlcNAcβ-sp4
	10A	Neu5Acα2-3Galβ1-3(Fucα1-4)GlcNAc
	10B	Neu5Acα2-3Galβ1-4(Fucα1-3)GlcNAc
	10C	Neu5Acα2-3Galβ1-3GlcNAcβ1-3Galβ1-4Glc
	10D	Galβ1-4(Fucα1-3)GlcNAcβ1-6(Neu5Acα2-6Galβ1-4GlcNAcβ1-3)Galb1-4Glc
	10E	Neu5Acα2-3Galβ1-3(Neu5Acα2-6)GalNAc
	10H	Neu5Acα2-6Galβ1-3GlcNAcβ1-3Galβ1-4(Fucα1-3)Glc
	10K	Neu5Acα2-3Galβ1-4GlcNAc
	10L	Neu5Acα2-6Galβ1-4GlcNAc
	10M	Neu5Acα2-3Galβ1-3GlcNAcβ1-3Galβ1-4Glc
	10N	Galβ1-3(Neu5Acα2-6)GlcNAcβ1-3Galβ1-4Glc
	10O	Neu5Acα2-6Galβ1-4GlcNAcβ1-3Galβ1-4Glc
	10P	Neu5Acα2-3Galβ1-3(Neu5Acα2-6)GlcNAcβ1-3Galβ1-4Glc
	11A	Neu5Acα2-3Galβ1-4Glc
	11B	Neu5Acα2-6Galβ1-4Glc
	11C	(Neu5Acα2-8Neu5Ac)n (n<50)
	11D	Neu5Acα2-6Galβ1-4GlcNAcβ1-2Manα1-6(Neu5Acα2-6Galβ1-4GlcNAcβ1-2Manα1-6)Manβ1-4GlcNAcβ1-4GlcNAc-Asn
	4	GalNAcα-sp0
	5	GalNAcα-sp3
	6	GalNAcβ-sp3

**Terminal GalNAc**	38	3-O-Su-GalNAcα-sp3	
	101	GalNAcα1-3GalNAcβ-sp3	
	102	GalNAcα1-3Galβ-sp3	
	103	GalNAcα1-3GalNAcα-sp3	
	104	GalNAcβ1-3Galβ-sp3	
	106	GalNAcβ1-4GlcNAcβ-sp3	
	192	GalNAcβ1-4(6-O-Su)GlcNAcβ-sp3	
	193	3-O-Su-GalNAcβ1-4GlcNAcβ-sp3	
	194	6-O-Su-GalNAcβ1-4GlcNAcβ-sp3	
	195	6-O-Su-GalNAcβ1-4-(3-O-Su)GlcNAcβ-sp3	
	196	3-O-Su-GalNAcβ1-4(3-O-Su)-GlcNAcβ-sp3	
	197	3,6-O-Su$_2$-GalNAcβ1-4GlcNAcβ-sp3	
	198	4,6-O-Su$_2$-GalNAcβ1-4GlcNAcβ-sp3	
	199	4,6-O-Su$_2$-GalNAcβ1-4-(3-O-Ac)GlcNAcβ-sp3	
	200	4-O-Su-GalNAcβ1-4GlcNAcβ-sp3	
	201	3,4-O-Su$_2$-Galβ1-4GlcNAcβ-sp3	
	202	6-O-Su-GalNAcβ1-4(6-O-Su)GlcNAcβ-sp3	
	204	4-O-Su-GalNAcβ1-4GlcNAcβ-sp2	
	238	GalNAcβ1-4Galβ1-4Glcβ-sp3	
	389	GalNAcβ1-3GalαGalβ1-4Glcβ-sp3	
**Terminal Glucose**	7	Glcα-sp3	
	9	Glcβ-sp3	
	46	6-H_2PO_3Glcβ-sp4	
	110	Glcα1-4Glcβ-sp3	
	11	Glcβ1-4Glcβ-sp4	
	112	Glcβ1-6Glcβ-sp4	
	240	(Glcα1-4)$_3$β-sp4	
	241	(Glcα1-6)$_3$β-sp4	
	390	(Glcα1-4)$_4$β-sp4	
	391	(Glcα1-6)$_4$β-sp4	
	492	(Glcα1-6)$_5$β-sp4	
	502	(Glcα1-6)$_6$β-sp4	
**GAG digests**	12A	Neocarratetraose-4^{1,3}-di-*O*-sulphate (Na^+)	
	12B	Neocarratetraose-4^{1}-*O*-sulphate (Na^+)	
	12C	Neocarrahexaose-2^{4},4^{1,3,5}-tetra-*O*-sulphate (Na^+)	
	12D	Neocarrahexaose-4^{1,3,5}-tri-*O*-sulphate (Na^+)	
	12E	Neocarraoctaose-4^{1,3,5,7}-tetra-*O*-sulphate (Na^+)	
	12F	Neocarradecaose-4^{1,3,5,7,9}-penta-*O*-sulphate (Na^+)	
	12G	ΔUA-2S → GlcNS-6S Na_4 (I-S)	
	12H	ΔUA → GlucNS-6S Na_3 (II-S)	
	12I	ΔUA → 2S-GlcNS Na_3 (III-S)	
	12J	ΔUA → 2S-GlcNAc-6S Na_3 (I-A)	
	12K	ΔUA → GlcNAc-6S Na_2 (II-A)	
	12L	ΔUA → 2S-GlcNAc Na_2 (III-A)	
	12M	ΔUA → GlcNAc Na (IV-A)	

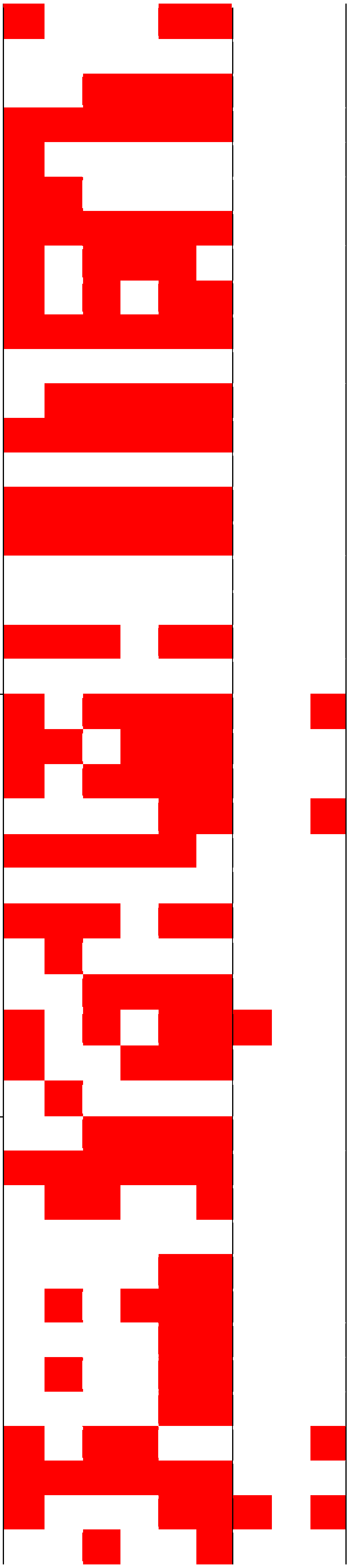

	12N	ΔUA → GalNAc-4S Na_2 (Δ Di-4S)									
	12O	ΔUA → GalNAc-6S Na2 (Δ Di-6S)	■	■	■		■	■			
	12P	ΔUA → GalNAc-4S,6S Na_3 (Δ Di-disE)	■	■		■	■	■			
	13A	ΔUA → 2S-GalNAc-4S Na_2 (Δ Di-disB)	■	■	■	■	■	■			
	13B	ΔUA → 2S-GalNAc-6S Na_3 (Δ Di-disD)	■	■	■		■				■
	13C	ΔUA → 2S-GalNAc-4S-6S Na_4 (Δ Di-tisS)		■			■	■			
	13D	ΔUA → 2S-GalNAc-6S Na_2 (ΔDi-UA2S)		■			■	■			
	13E	ΔUA → GlcNAc Na (Δ Di-HA)	■	■	■	■	■	■			
**GAGs**	13F	(GlcAβ1-3GlcNAcβ1-4)n (n=4)					■	■	■		
	13G	(GlcAβ1-3GlcNAcβ1-4)n (n=8)					■	■			
	13H	(GlcAβ1-3GlcNAcβ1-4)n (n=10)							■	■	
	13I	(GlcAβ1-3GlcNAcβ1-4)n (n=12)					■	■	■		
	13J	(GlcA/IdoAα/β1-4GlcNAcα1-4)n (n=200)	■	■	■	■	■	■	■		■
	13K	(GlcA/IdoAβ1-3(±4/6S)GalNAcβ1-4)n (n<250)	■	■	■	■	■	■			
	13L	((±2S)GlcA/IdoAα/β1-3(±4S)GalNAcβ1-4)n (n<250)			■		■	■			
	13M	(GlcA/IdoAβ1-3(±6S)GalNAcβ1-4)n (n<250)		■			■				
	13N	(GlcAβ1-3GlcNAcβ1-4)n (n=4)	■	■	■	■	■	■			
	13O	(GlcAβ1-3GlcNAcβ1-4)n (n=6)	■	■	■	■	■	■			
	13P	(GlcAβ1-3GlcNAcβ1-4)n (n=8)			■		■	■	■		
	14A	(GlcAβ1-3GlcNAcβ1-4)n (n=10)									
	14B	(GlcAβ1-3GlcNAcβ1-4)n (n=12)	■				■	■		■	
	14C	(GlcAβ1-3GlcNAcβ1-4)n (n=14)	■	■		■		■			
	14D	(GlcAβ1-3GlcNAcβ1-4)n (n=16)	■	■	■		■	■			
	14E	(GlcAβ1-3GlcNAcβ1-4)-30,000da					■	■			
	14F	(GlcAβ1-3GlcNAcβ1-4)-107,000da		■	■	■	■	■			■
	14G	(GlcAβ1-3GlcNAcβ1-4)-190,000da	■	■	■	■	■	■			■
	14H	(GlcAβ1-3GlcNAcβ1-4)-220,000da	■	■	■	■	■	■	■	■	■
	14I	(GlcAβ1-3GlcNAcβ1-4)-1,600,000da	■		■	■	■	■			
	14J	Heparan Sulfate 5mg/ml	■	■		■	■	■			
	14K	(Glcβ1-3Glcβ1-3)n	■		■	■	■	■			
**Other**	14	GlcN(Gc)β-sp4			■	■	■				
	15	$HOCH_2(HOCH)_4CH_2NH_2$				■	■				
	20	Rhaα-sp3				■	■	■			
	44	GlcAα-sp3		■							
	45	GlcAβ-sp3	■	■		■		■			
	164	GlcAβ1-3GlcNAcβ-sp3	■	■	■	■	■	■			
	165	GlcAβ1-3Galβ-sp3	■		■	■	■	■			
	166	GlcAβ1-6Galβ-sp3									
	625	$(GlcA\beta1\text{-}4GlcNAc\beta1\text{-}3)_8$-$NH_2$-ol	■	■	■	■	■	■			

Red represents binding, which is defined as positive if the average fluorescence intensity of the 4 repeat glycan spots is greater than one-fold above the adjusted background (average of the slide background plus three standard deviations) in three independent replicates (Students T-test $p<0.001$). Strains used: **MC58**, Cap+, L3 LOS, pilin+, Opc+; **MC58Δ*pilE***, Cap+, L3 LOS, pilin-, Opc+; **Ȼ3**, Cap-, L3 LOS, pilin+, Opc+; **Ȼ3Δ*pilE***, Cap-, L3 LOS, pilin-, Opc+; **Ȼ2**, Cap-, L8 LOS, pilin+, Opc+; **Ȼ9**, Cap-, L8 LOS, pilin+, Opc-.

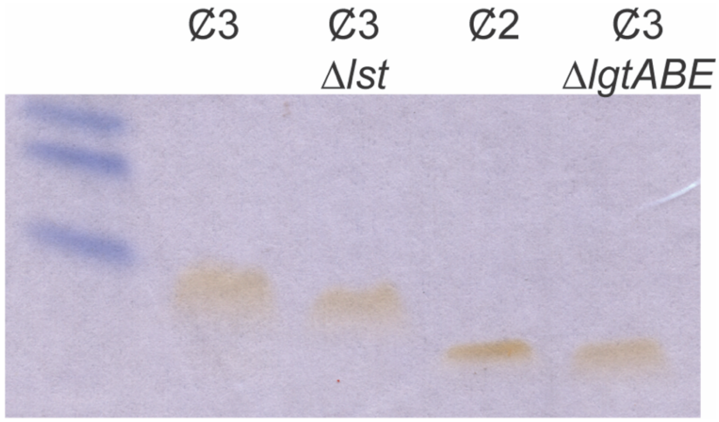

Figure S1. Silver-stained Tricine-SDS-PAGE gel showing LOS purified from *N. meningitidis* Ȼ3, Ȼ3*Δlst*, Ȼ2 and Ȼ3*ΔlgtABE*strains. 5µg of LOS loaded per lane.

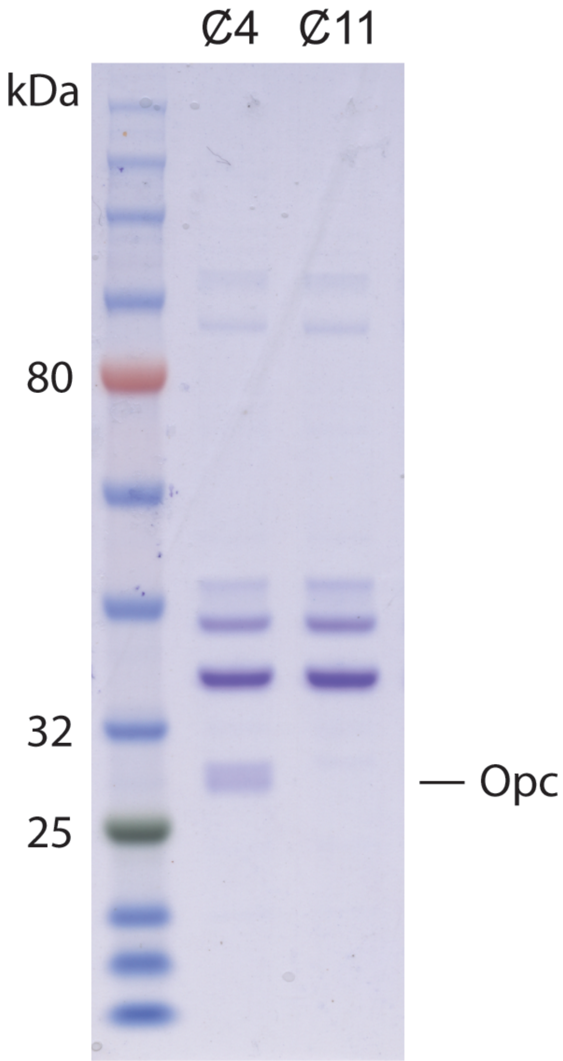

Figure S2. Coomassie Blue stained Bis-Tris SDS-PAGE gel showing outer membrane preparations from the Opc expressing strain (Ȼ4) and the isogenic Opc mutant strain (Ȼ11). 7.5µg total protein loaded per lane.

Supplementary dataset S1: (i) Representative sensorgrams of the glycan interactions with the Opc+ outer membrane preparations (OMPs). **(ii)** Sigmoidal curves from ITC analysis of Opc-glycan interactions (calculated from Opc+ using the double blank subtraction of the heats of injections from glycan into PBS, and from glycan into Opc- OMP). Opc interactions with **(A)** sialyl LewisX, **(B)** α2-6-sialyllactose, **(C)** blood group H antigen, **(D)** lacto-N-neotetraose, **(E)** chondroitin-6-sulfate, and **(F)** asialo GM1 are shown.

A. sialyl LewisX

(i)

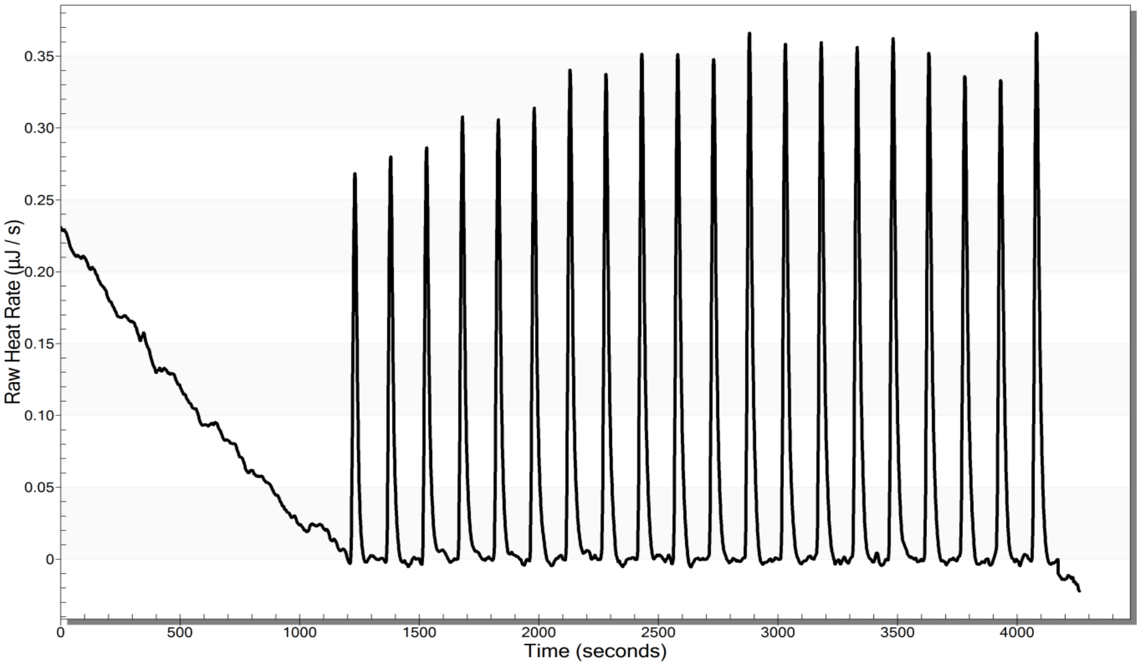

(ii)

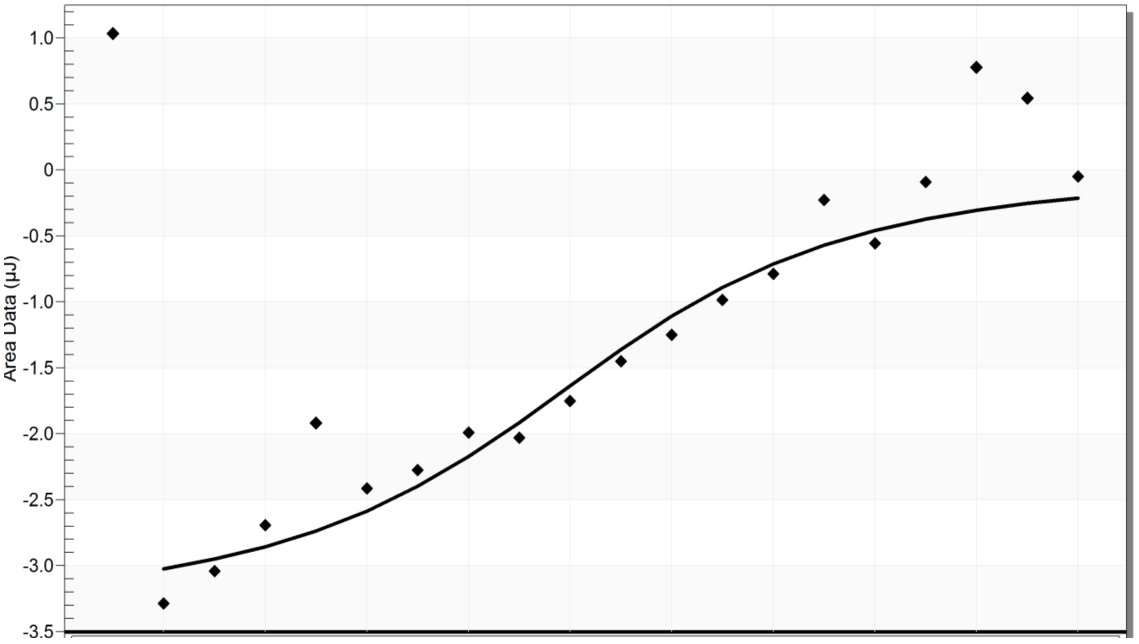

B. **α2-6-sialyllactose**

(i)

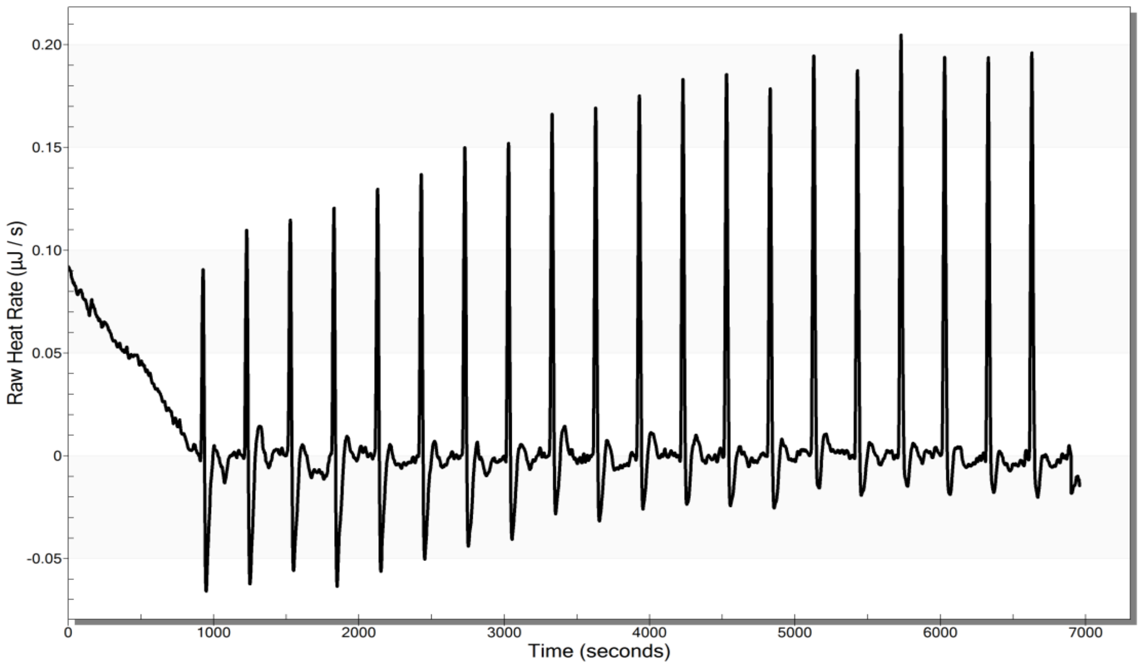

(ii)

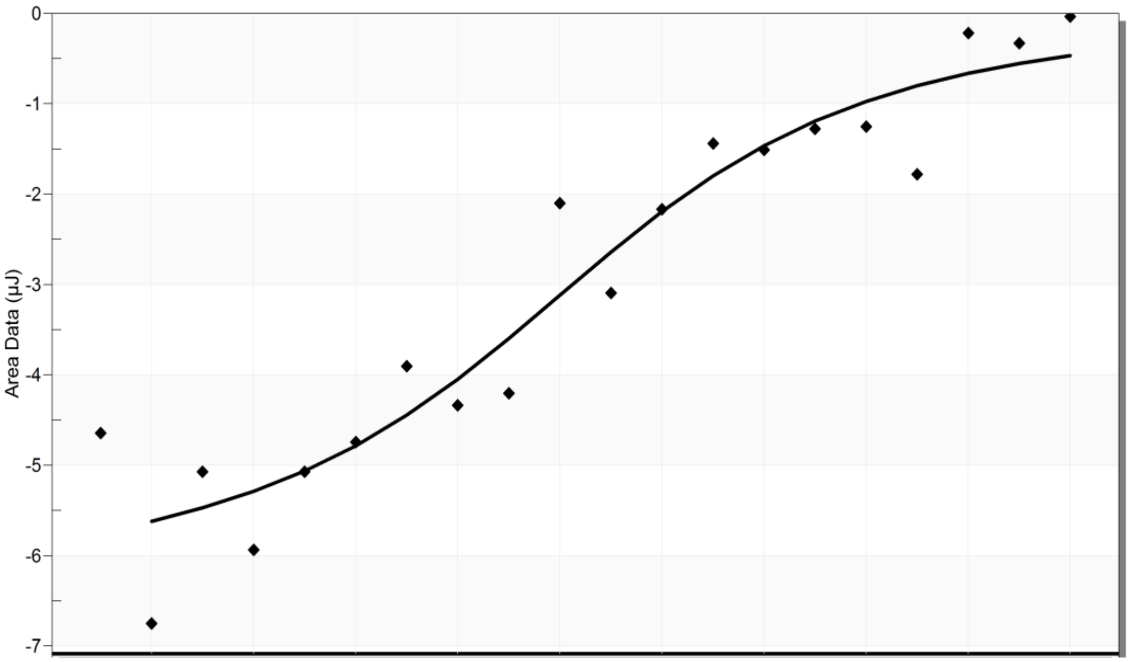

C. blood group H antigen

(i)

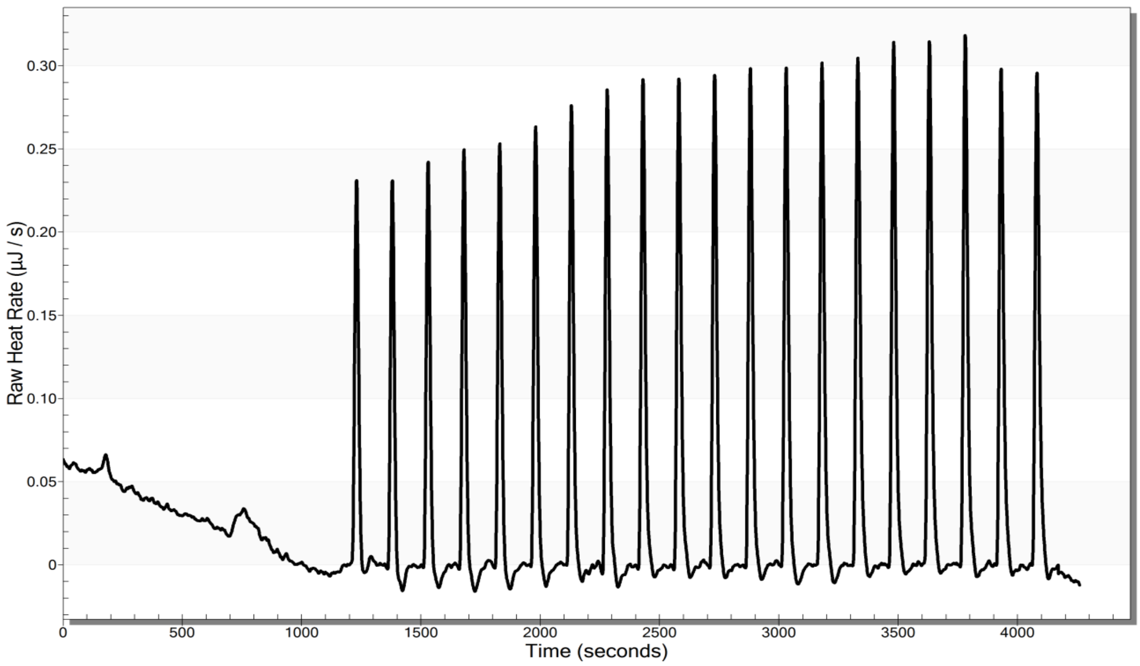

(ii)

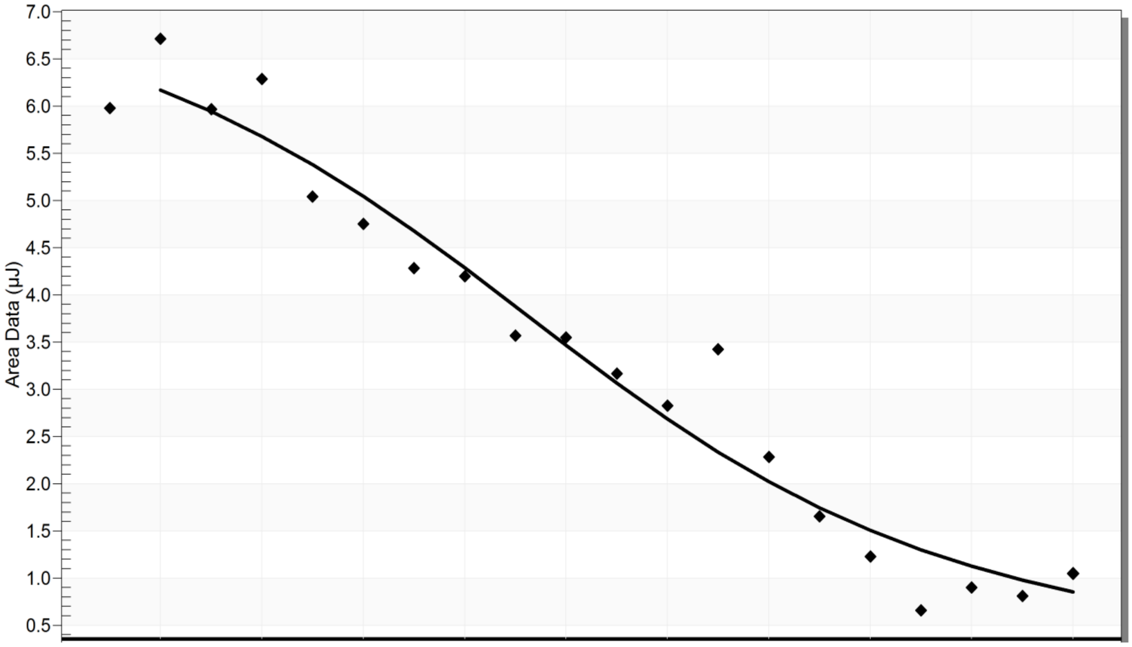

D. lacto-*N*-neotetaraose

(i)

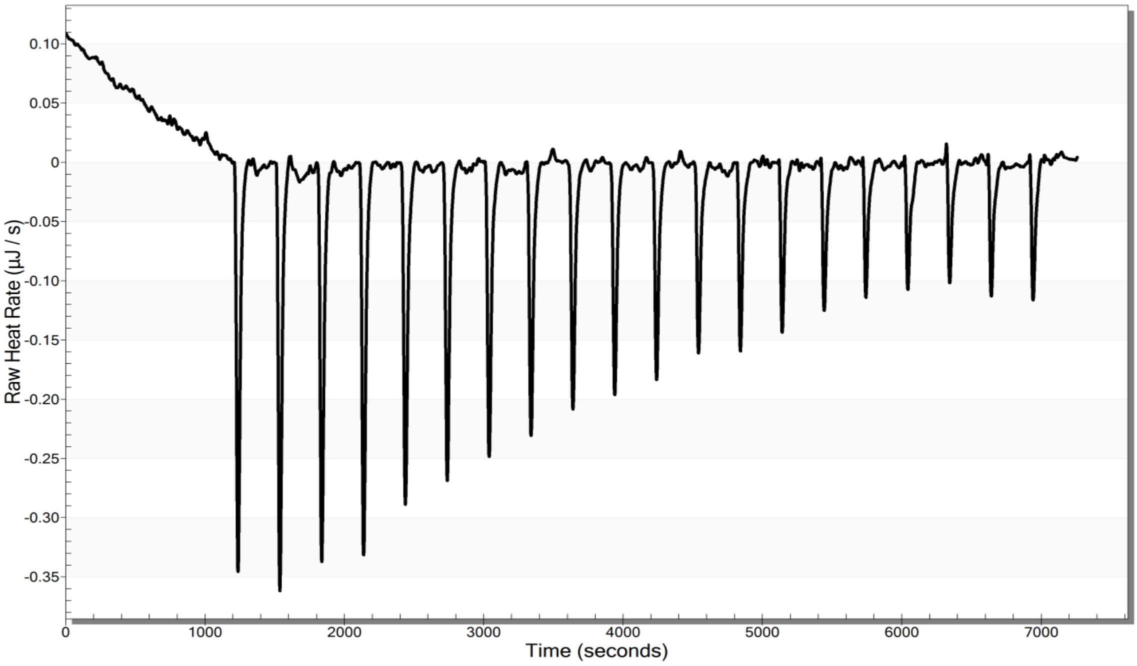

(ii)

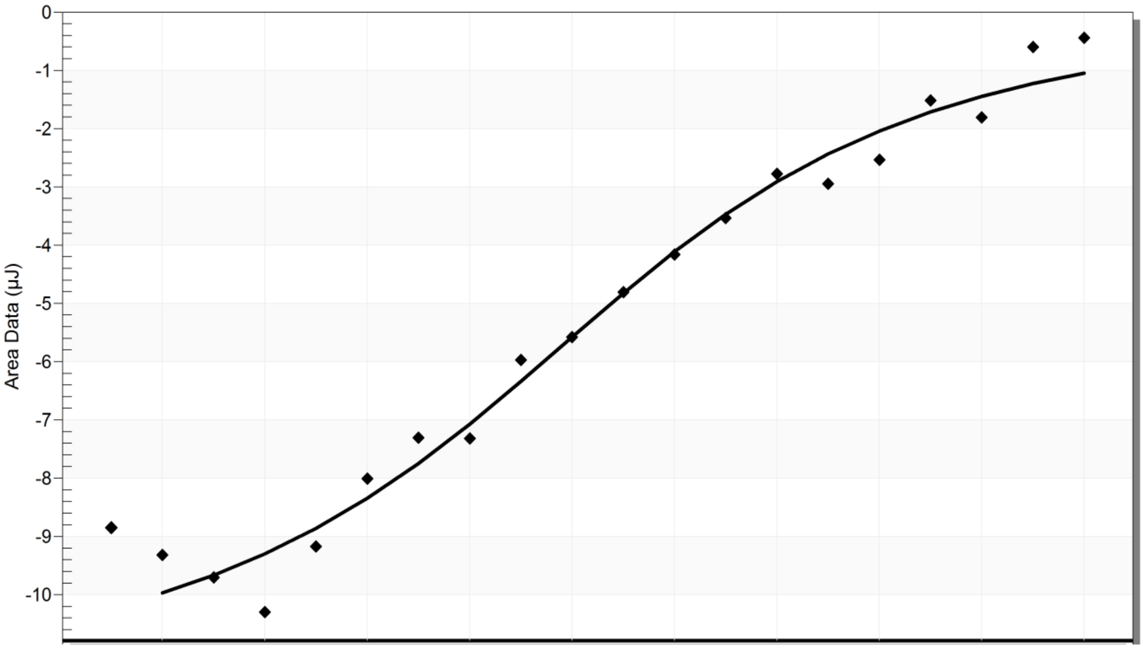

E. chondroitin-6-sulfate

(i)

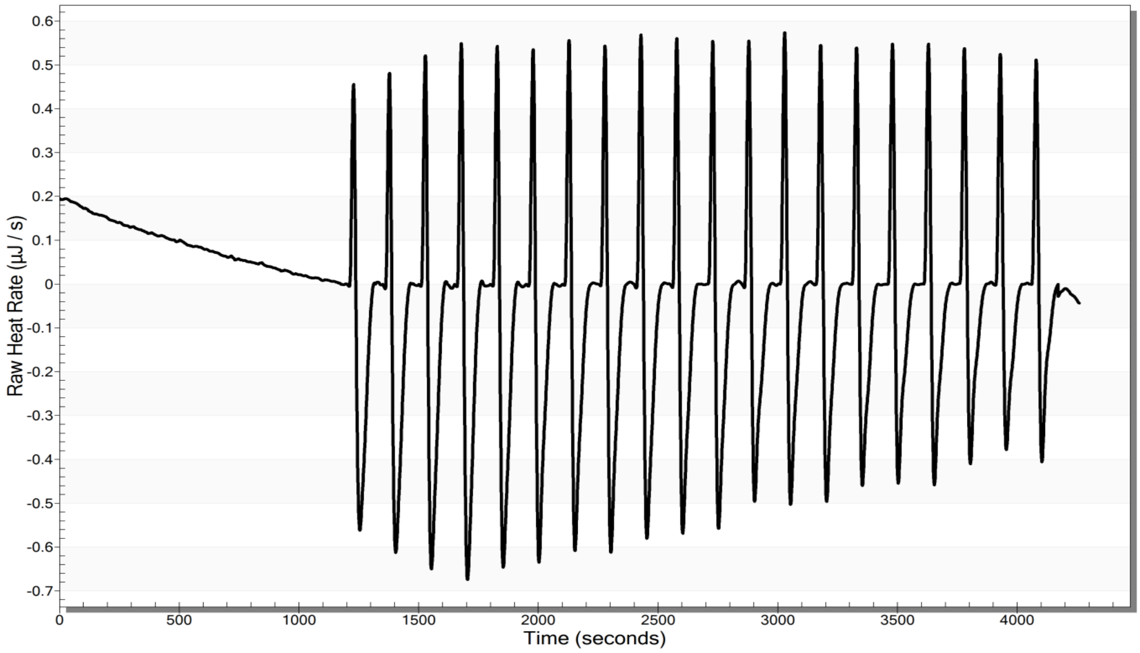

(ii)

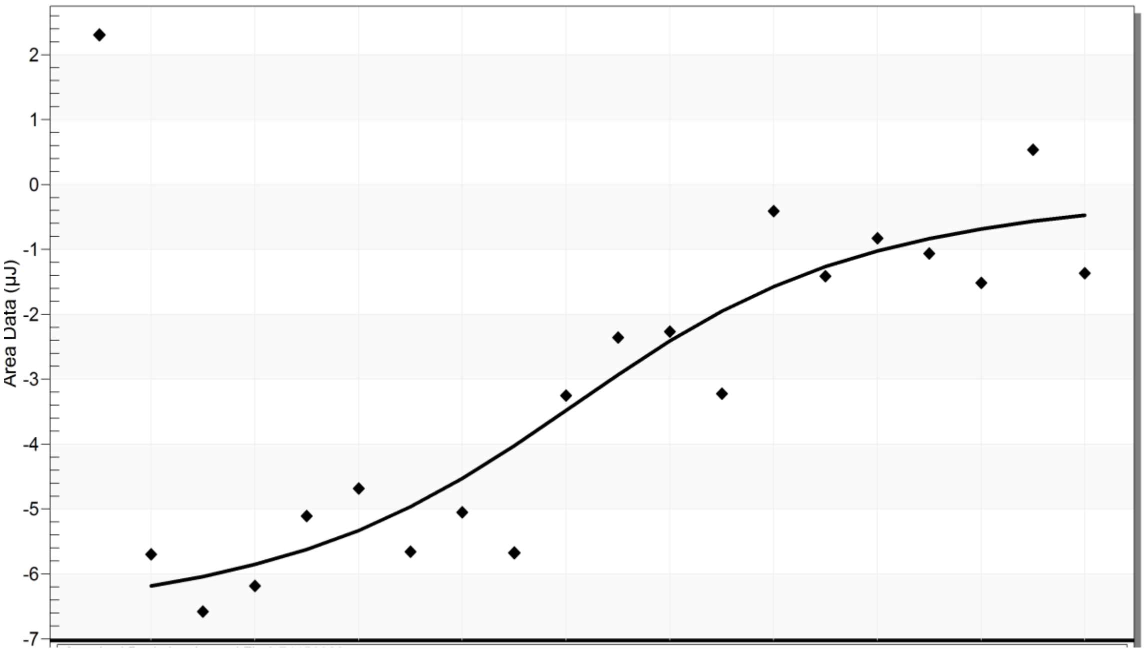

F. asialo GM1

(i)

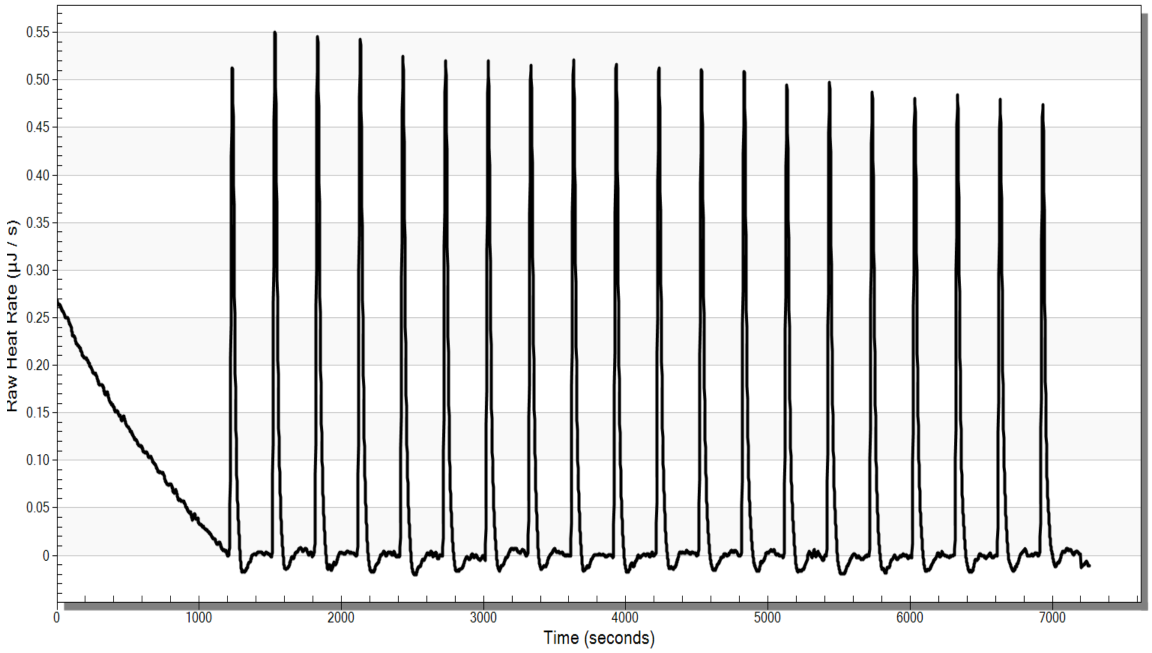

(ii)

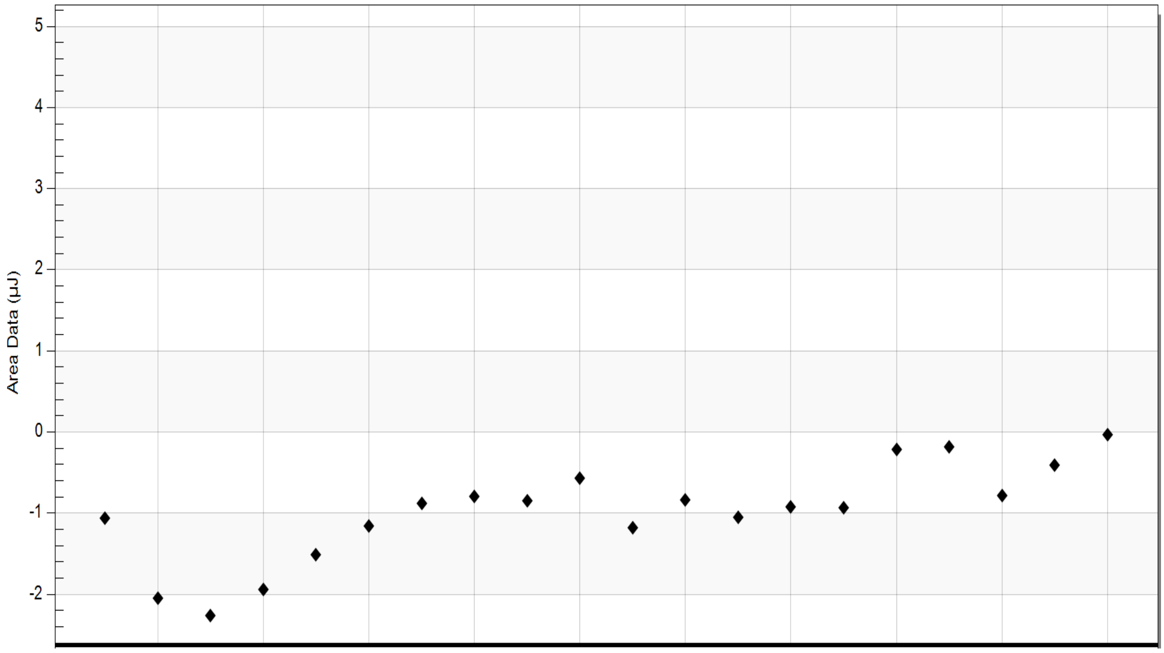

Supplementary dataset S2. Representative sensograms from surface plasmon resonance (SPR) analysis of lipooligosaccharide (LOS)-glycan interactions. (**A**) L3 LOS, (**B**) Δ*lst* LOS, (**C**) L8 LOS and (**D**) Δ*lgtABE* LOS, with **(i)** lacto-*N*-biose I, (**ii**) TF antigen, (**iii**) α1-3 galactobiose, (**iv**) colominic acid and (**v**) heparin. Graphs show response units (y-axis) over time (x- axis).

(A)

i) L3 LOS – lacto-*N*-biose I

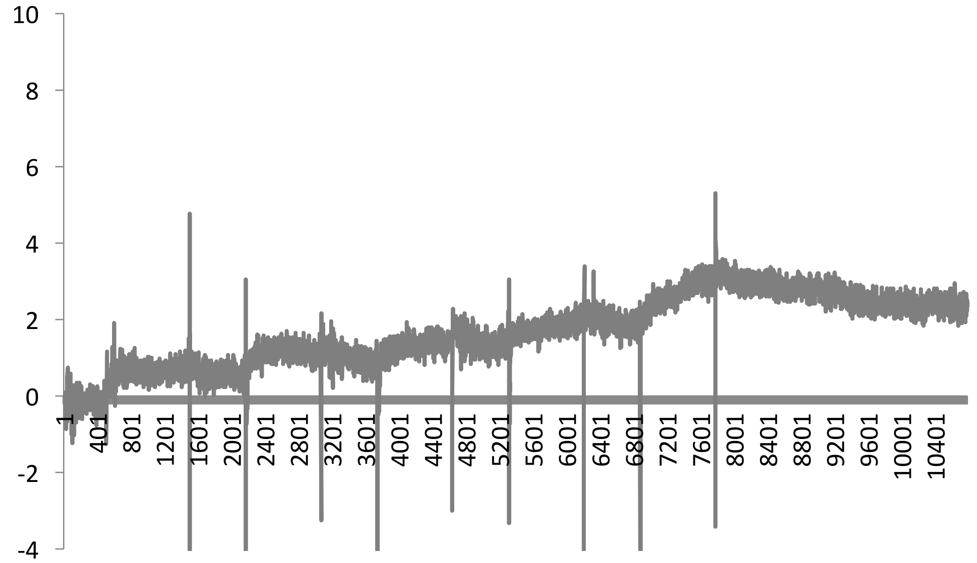

ii) L3 LOS – TF antigen

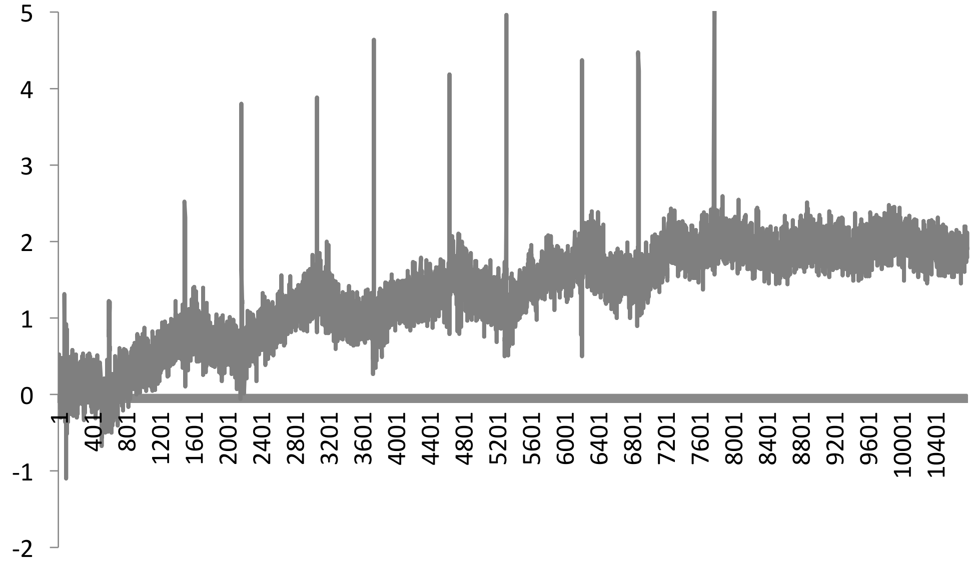

iii) L3 LOS – α1-3 galactobiose

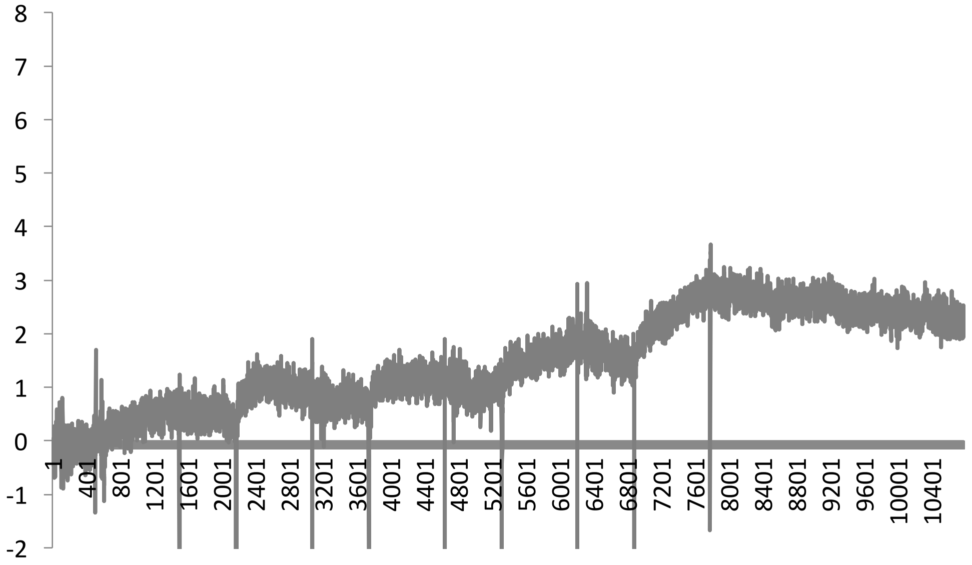

iv) L3 LOS – colominic acid

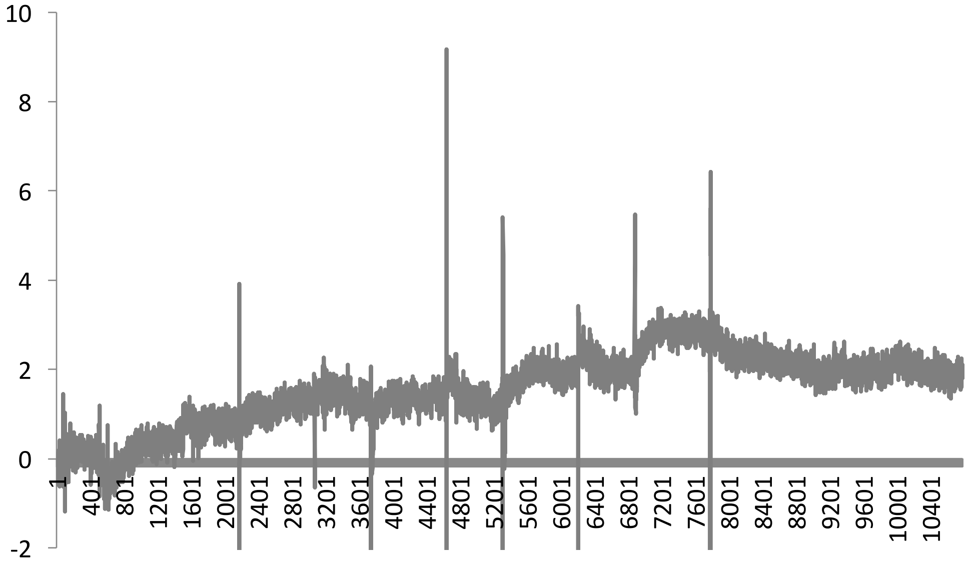

v) L3 LOS – heparin

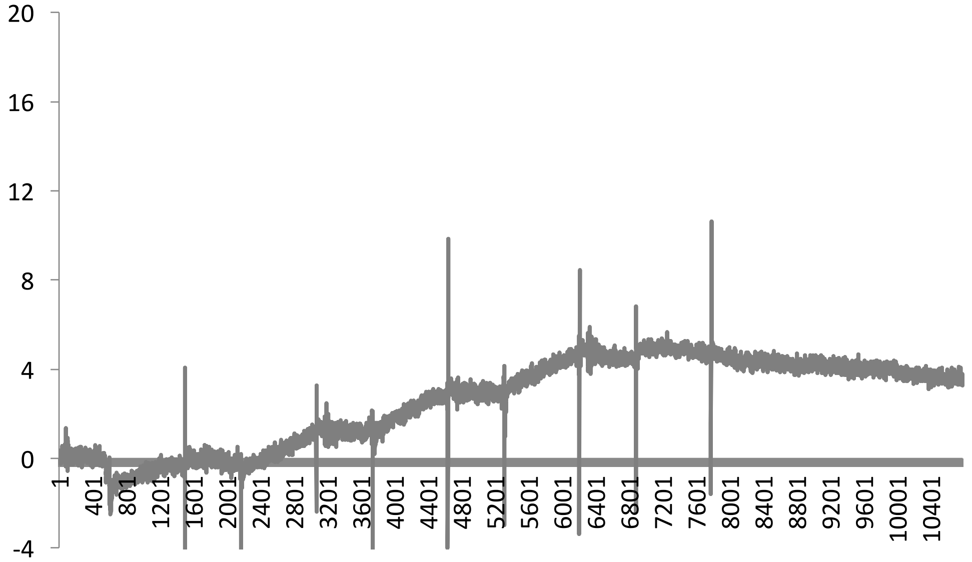

(B)

i) Δ*lst* LOS – lacto-*N*-biose I

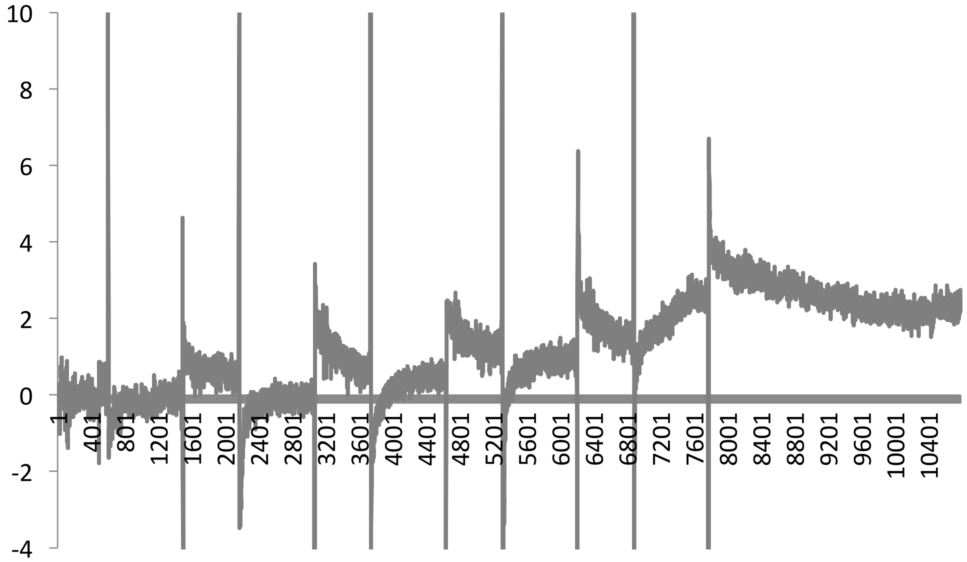

ii) Δ*lst* LOS – TF antigen

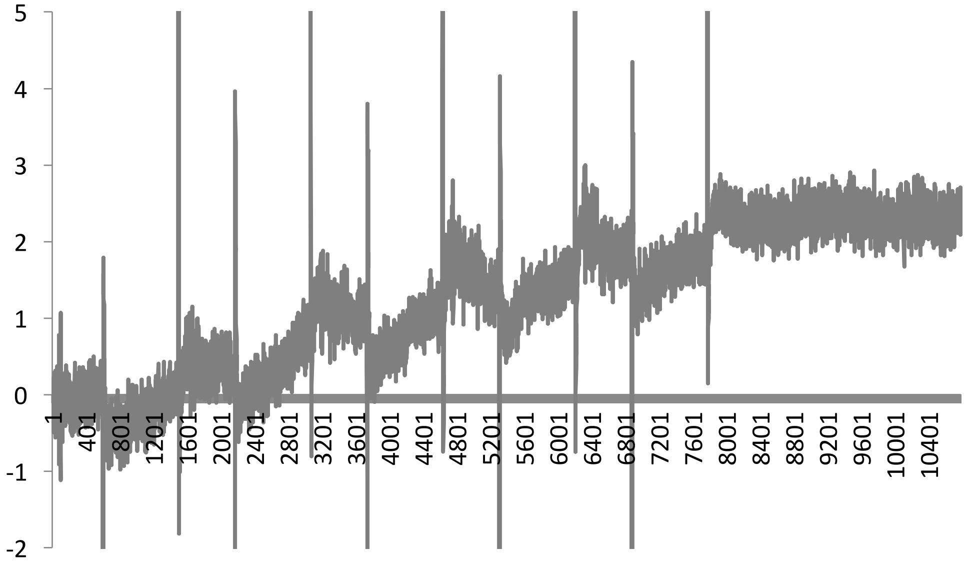

iii) Δ*lst* LOS – α1-3 galactobiose

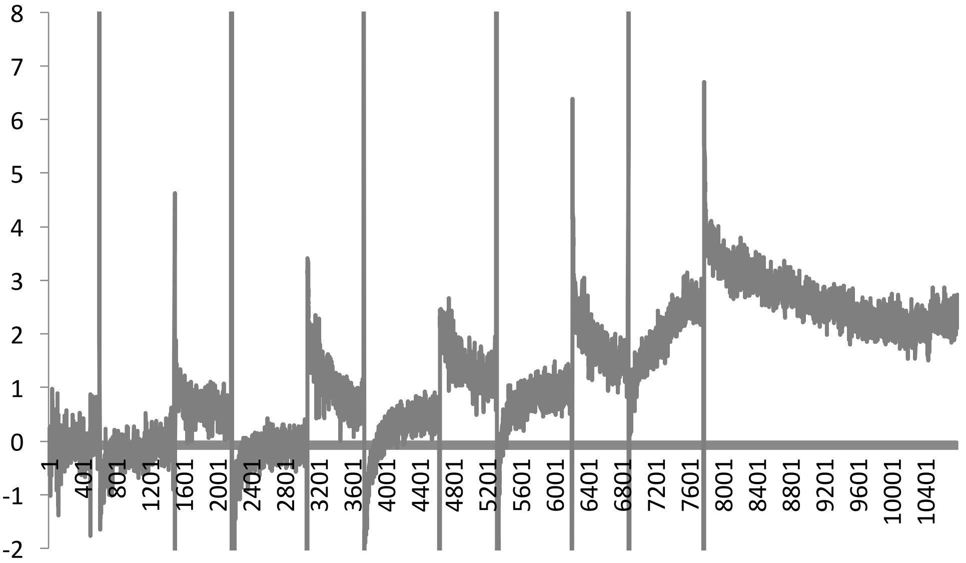

iv) Δ*lst* LOS – colominic acid

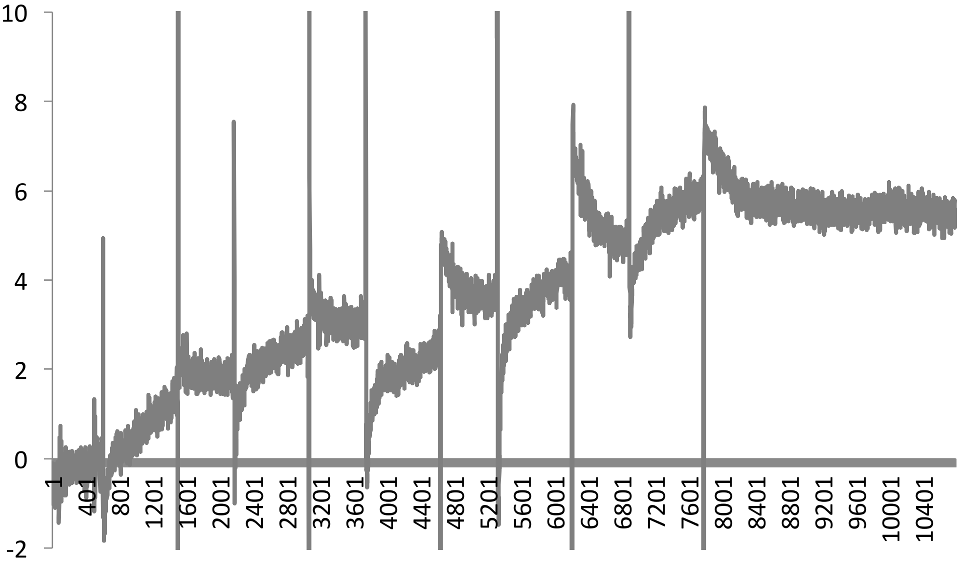

v) Δ*lst* LOS – heparin

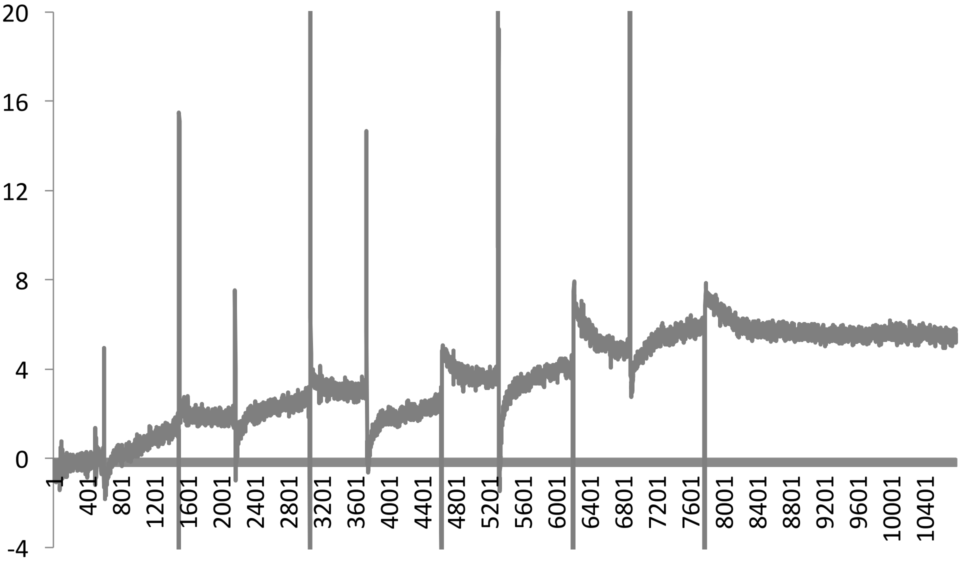

(C)

i) L8 LOS – lacto-*N*-biose I

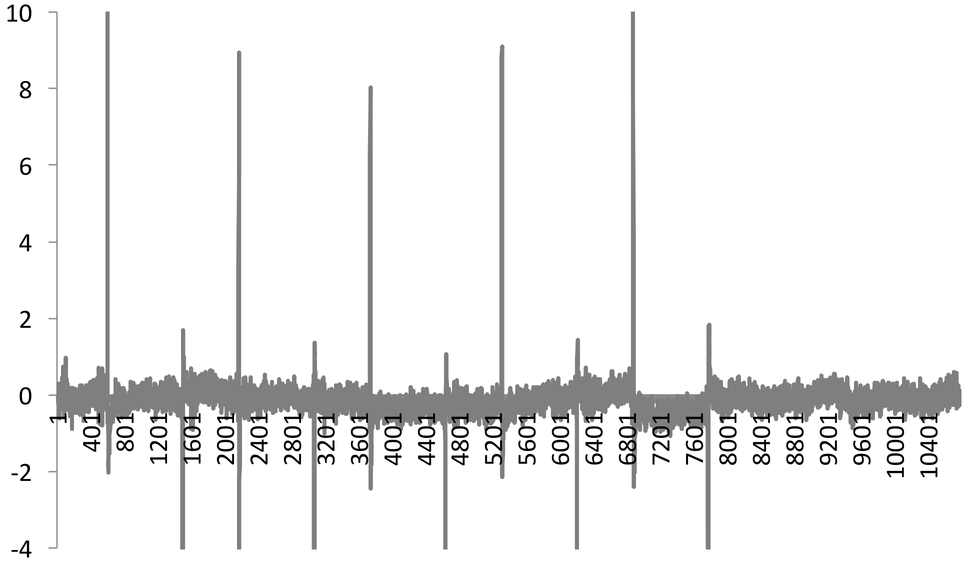

ii) L8 LOS – TF antigen

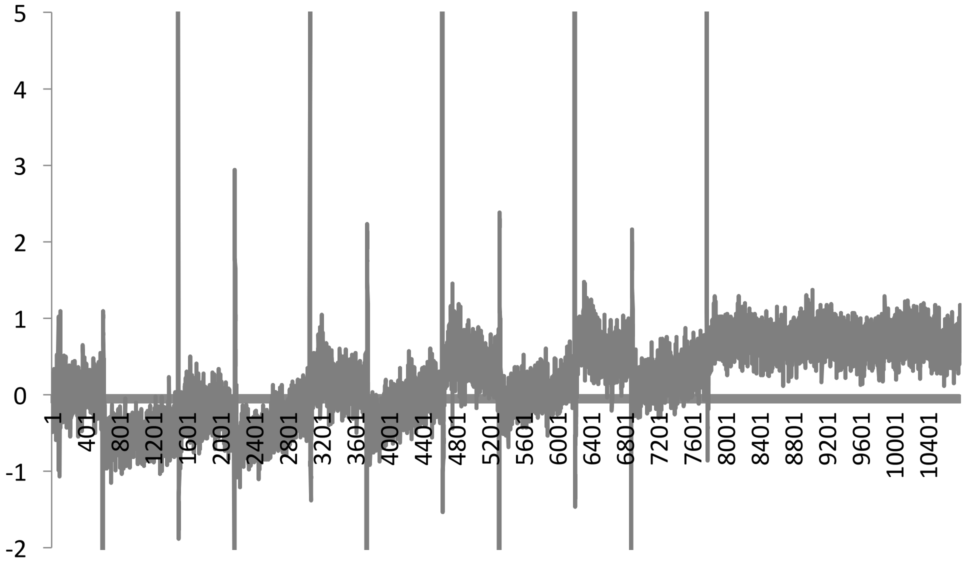

iii) L8 LOS – α1-3 galactobiose

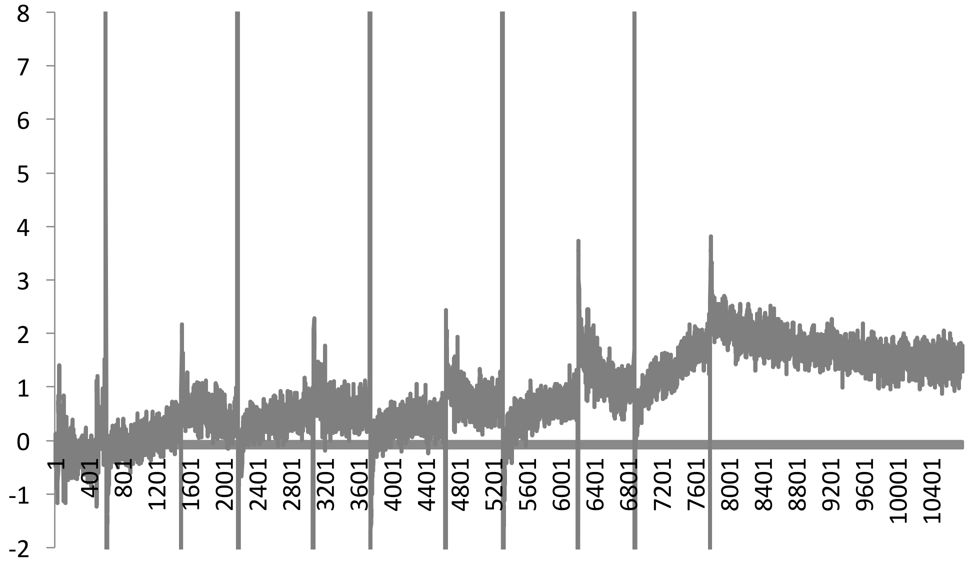

iv) L8 LOS – colominic acid

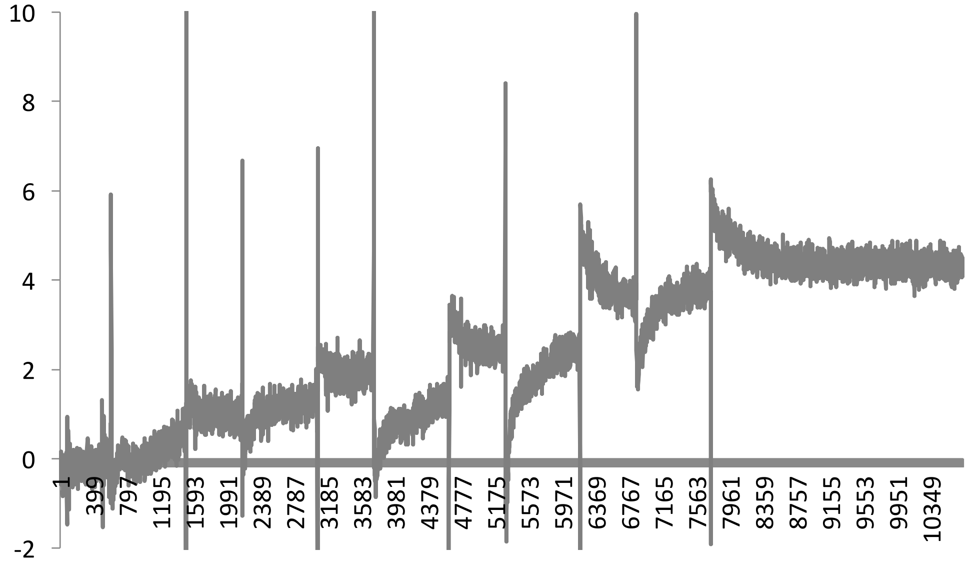

v) L8 LOS – heparin

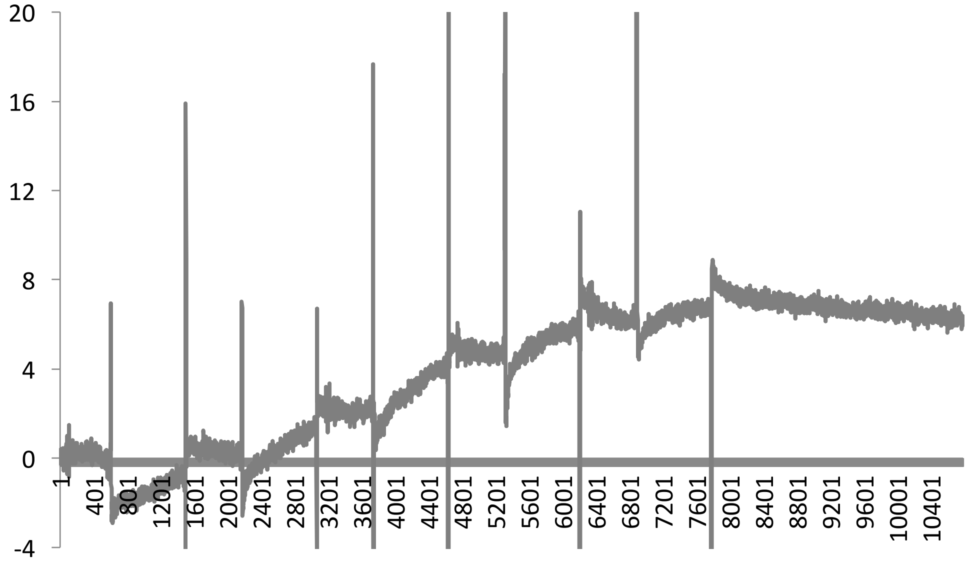

(D)

i) Δ*lgtABE* LOS – lacto *N*-biose I

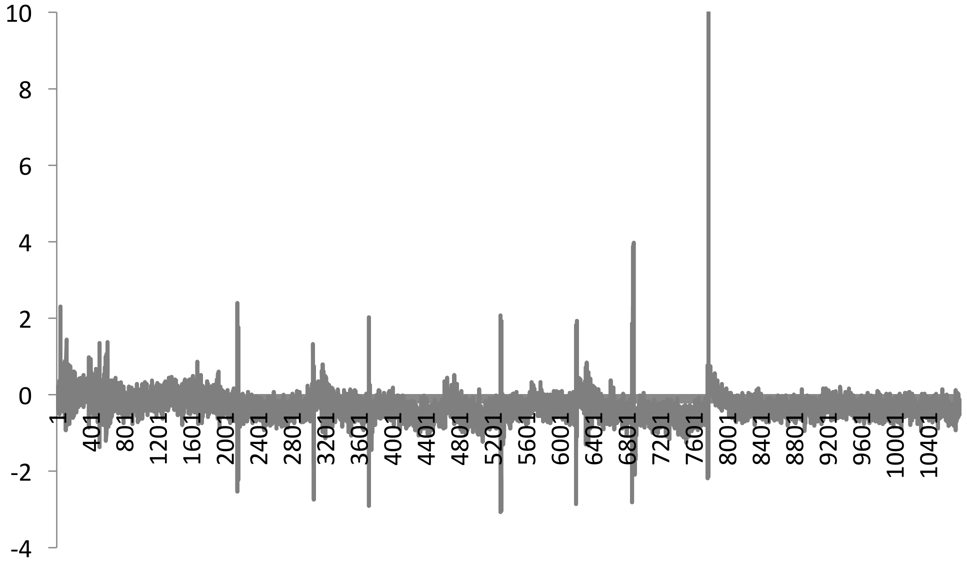

ii) Δ*lgtABE* LOS – TF antigen

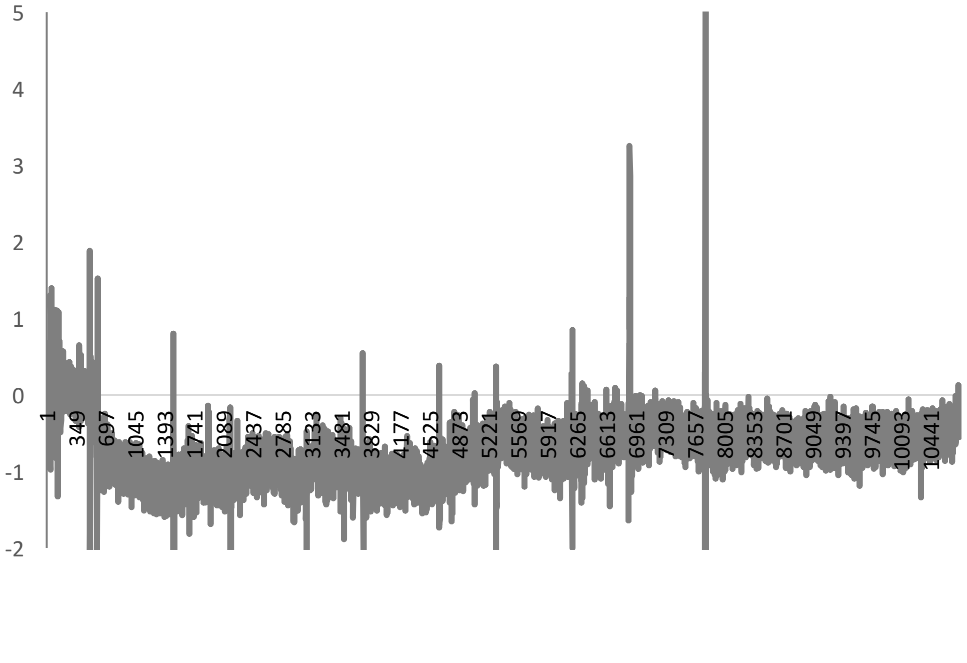

iii) Δ*lgtABE* LOS – α1-3 galactobiose

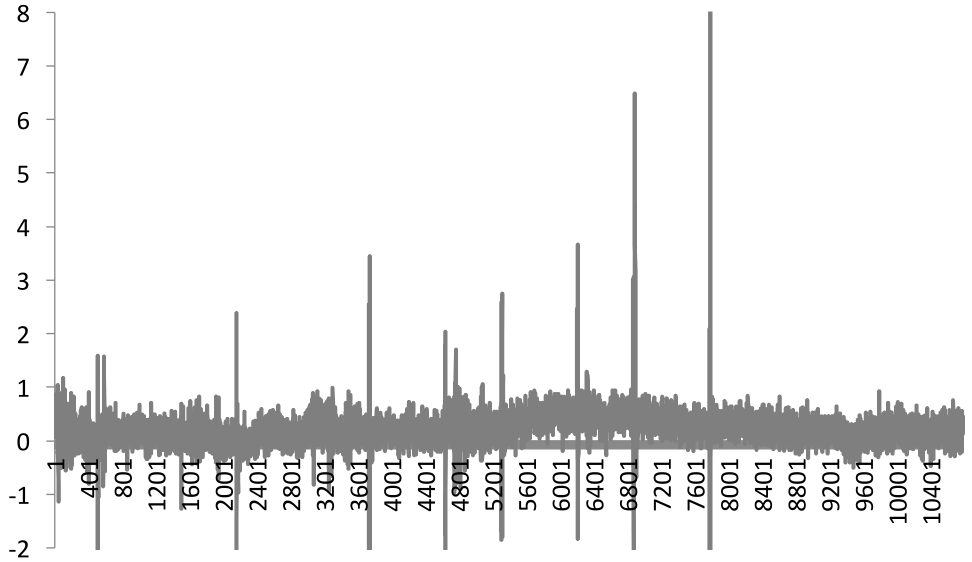

iv) Δ*lgtABE* LOS – colominic acid

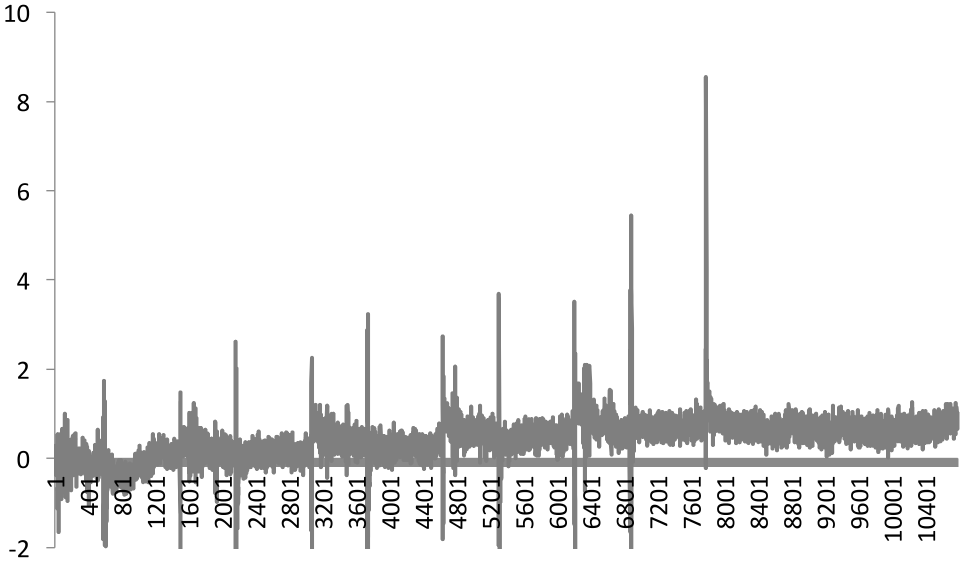

v) Δ*lgtABE* LOS – heparin

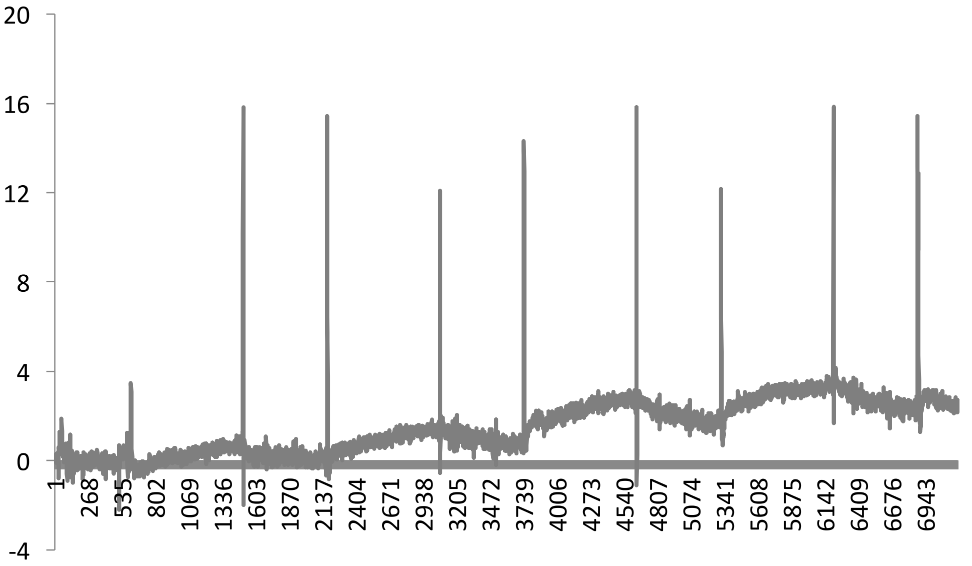

Supplement: Supplementary file 1 — Supplementary information [file 41598_2017_5894_MOESM1_ESM.pdf]
